# Exploring Predictive Factors for Bulevirtide Treatment Response in Hepatitis Delta-Positive Patients

**DOI:** 10.3390/biomedicines13020280

**Published:** 2025-01-23

**Authors:** Verdiana Zulian, Leonidas Salichos, Chiara Taibi, Silvia Pauciullo, Levi Dong, Gianpiero D’Offizi, Elisa Biliotti, Alessia Rianda, Luigi Federici, Angela Bibbò, Martina De Sanctis, Fiona McPhee, Anna Rosa Garbuglia

**Affiliations:** 1 Virology Laboratory, National Institute for Infectious Diseases Lazzaro Spallanzani IRCCS, 00149 Rome, Italy; silvia.pauciullo@inmi.it (S.P.); luigi.federici@inmi.it (L.F.); angela.bibbo@inmi.it (A.B.); desanctis.1635418@studenti.uniroma1.it (M.D.S.); annarosa.garbuglia@inmi.it (A.R.G.); 2 Department of Biological and Chemical Sciences, New York Institute of Technology, New York, NY 10023, USA; lsalicho@nyit.edu (L.S.); ldong06@nyit.edu (L.D.); 3 Infectious Diseases and Hepatology Unit, National Institute for Infectious Diseases Lazzaro Spallanzani IRCCS, 00149 Rome, Italy; chiara.taibi@inmi.it (C.T.); gianpiero.doffizi@inmi.it (G.D.); elisa.biliotti@inmi.it (E.B.); alessia.rianda@inmi.it (A.R.); 4 Independent Researcher, North Devon EX31, UK; mcpheef@gmail.com

**Keywords:** HDV, bulevirtide, antiviral treatment, Anti-HBc IgG, genetic variability

## Abstract

**Background:** Hepatitis delta virus (HDV) infection represents the most severe form of viral hepatitis and is a significant global health challenge. Bulevirtide (BLV) is a novel therapeutic treatment that has resulted in variable response rates in HBV/HDV-coinfected patients. We evaluated clinical, virological, and polymorphic factors for the purpose of predicting BLV treatment success. **Methods:** Thirty HBV/HDV-coinfected patients received BLV monotherapy (2 mg/day) for 24 to 48 weeks. Baseline (BL) serum samples were collected to assess clinical parameters and virological markers (HDV RNA, HBV DNA, HBsAg, HBcrAg, anti-HBc IgG) at treatment weeks 24 (TW24) and 48 (TW48). Additionally, full-genome HDV sequencing and a phylogenetic analysis were performed. Finally, analyses of the HDAg protein sequence and HDV RNA secondary structure were conducted to evaluate potential associations with treatment response. **Results:** A significant reduction in HDV RNA levels was observed at TW48, with a virological response (HDV RNA undetectable or ≥2 Log decline from BL) achieved by 58% of patients. Median BL levels of anti-HBc IgG were significantly different between virological responders (39.3 COI; interquartile range [IQR] 31.6–47.1) and virological non-responders (244.7 COI; IQR 127.0–299.4) (*p* = 0.0001). HDV genotype 1e was predominant across the cohort, and no specific HDAg polymorphisms predicted the response. However, secondary structure analysis of HDV RNA revealed that a specific pattern of internal loops in the region 63–100 nucleotides downstream of the editing site may influence treatment response by impacting editing efficacy. **Conclusions:** This study revealed key factors influencing BLV efficacy in HBV/HDV coinfection. Lower baseline anti-HBc IgG levels strongly correlated with a positive virological response, suggesting that the liver’s inflammatory state affects treatment success. Additionally, the analysis of HDV RNA secondary structure in patients receiving BLV treatment revealed a higher editing efficiency in virological responders, highlighting areas for further research.

## 1. Introduction

Hepatitis delta virus (HDV) is a defective RNA virus that is the sole member of the Deltavirus genus in the *Kolmioviridae* family [1]. This satellite RNA virus requires the hepatitis B virus (HBV) surface protein (HBsAg) to produce infectious virions and propagate [2]. For viral entry into hepatocytes, HDV utilizes the sodium taurocholate cotransporting polypeptide (NTCP), a receptor that binds HBsAg. For replication, HDV lacks its own polymerase and instead exploits cellular RNA polymerase II. HDV represents a relevant contributor to the global burden of liver disease. It can infect hepatocytes either simultaneously with HBV, leading to a coinfection with relatively better clinical outcome, or as a superinfection in individuals with chronic HBV infection. Both acute and chronic infections are characterized by the suppression of HBV DNA [3]. Chronic HDV infection progresses to cirrhosis within an average of five years and accounts for 18% of cases of cirrhosis and 20% of hepatocellular carcinoma (HCC) among HBV-infected patients [4]. The risk of developing HCC in HDV-positive patients is three times higher than in HBV-monoinfected patients [5]. Furthermore, a six-fold increase in liver cancer risk has been observed in HDV-HBV-coinfected patients compared to HBV-monoinfected patients [6]. For these reasons, HDV infection represents the main cause of liver transplantation in Europe [7]. In particular, among HIV-positive patients, liver-related mortality attributable to HIV-HDV coinfection has surpassed that associated with HBV or HCV monoinfection, owing to the availability of highly effective treatments for HBV and HCV [8,9]. In 2022, the global population chronically infected with HBV was estimated to be approximately 258 million [10,11]. The prevalence of HDV among HBV-infected individuals is 5–15%, corresponding to 14–26 million cases [4,12,13].

The HDV virion is encapsulated by small, medium, and large HBsAg and contains a RNP made up of a circular single-stranded, negative-sense RNA genome of around 1.68 kb and two isoforms of the only encoded HDV protein, HDAg [14]. One isoform is the large HDAg (L-HDAg, 27 kDa), essential for viral particle assembly [15], and the other is the small HDAg (S-HDAg, 24 kDa), crucial for viral replication and RNA translocation [16].

HDV is classified into eight genotypes and multiple subtypes, with genetic divergence among genotypes as high as 35–40% [17,18]. HDV genotype 1 is the most prevalent in Europe and North America [13], genotype 2 is mostly detected in Eastern Europe and Asia, genotype 3 is exclusively in South America (especially in the Amazon Basin), and genotype 4 is detected in China and Taiwan. Genotypes 5 through 8 circulate in Africa and are occasionally detected in Europe [13,17]. Genotype 8 also circulates in Brazil [19].

Although HBV is essential in the HDV replication cycle, drugs targeting HBV DNA polymerase, such as nucleoside analogues (NUCs), are ineffective against HDV, as the virus uses cellular polymerases for replication. Until recently, the only treatment options included interferon-alpha or pegylated interferon-alpha-2a (Peg-IFNα), sometimes combined with ribavirin, for 48 weeks. These treatments were only beneficial to small subsets of patients, with only ~20% achieving sustained virological response (SVR) [20]. In a 5-year follow-up study, 56% of patients who achieved SVR with Peg-IFNα treatment, with or without adefovir dipivoxil, experienced virological relapse after therapy discontinuation [21].

More recently, researchers at Heidelberg University in Germany synthetized a large lipopeptide, bulevirtide (BLV), which mimics a 47-amino-acid sequence of the hepatitis B surface antigen (HBsAg) preS1 region and competes for binding to the HDV receptor, NTCP, thereby blocking the entry of HBV and HDV [22,23].

BLV is generally well tolerated in patients treated for up to 144 weeks, and viral response (undetectable HDV RNA or ≥2 log10 IU/mL from baseline [BL]) has been shown to improve with longer treatment durations; 50% of patients achieved undetectable HDV RNA at week 144 [24,25,26,27]. For those patients who do not respond to this treatment (HDV RNA decline ≤1 Log IU/mL at the respective study endpoints), an understanding of the parameters impacting response remains elusive. Identifying factors that influence viral kinetics and treatment outcomes, such as HDV genotypes/subtypes, viral resistance, or host factors, is crucial.

In this study, we evaluated clinical and virological parameters, including HBsAg, HBcrAg, HBcAb, HDV RNA viral load, and HDV polymorphisms, that might predict the success of BLV therapy in HBV/HDV-coinfected patients.

## 2. Materials and Methods

### 2.1. Study Population

Thirty patients with chronic HBV/HDV coinfection treated with BLV were enrolled in this study. All patients received BLV monotherapy (2 mg/day) for at least 24 weeks. Specifically, 6 patients received 24 weeks of treatment, and 24 patients received 48 weeks of treatment at the time of analysis.

Twenty-one patients had a Metavir score = F4, while 7 patients had a score ≤ F3 at baseline (BL). Three patients tested positive for anti-HCV antibodies, although they were negative for HCV RNA for 10 years.

Serum samples were collected from all patients at BL and at treatment weeks (TW) 4, TW16, TW24, and TW48 for clinical and virological evaluation. Serological markers (HDV IgM, IgG, HBsAg, HBcAg, HBcrAg) were tested at all time points, alongside ALT, AST, albumin, bile acids, total bilirubin, and platelet count. Meanwhile, HBeAg, anti-HBeAg, and anti-core IgM were only tested at BL.

The HDV genotype and subtype were evaluated by sequencing.

The full-length HDV genome was isolated from BL samples from 24 patients who completed 48 weeks of treatment. Isolated genomes from BL samples were sequenced for HDAg polymorphism assessment. Additionally, the HDV antigenomic RNA secondary structure was determined using full-length HDV genome sequences isolated from BL samples.

All patients continued to receive their prescribed HBV NUC treatment.

The local ethics committee of Regione Lazio approved the study protocols (ethical approval number 97/2023). Written informed consent was obtained from all patients who participated in this study. All experiments were performed in accordance with the Declaration of Helsinki.

A virological response was defined as a ≥2 Log decline in HDV RNA or when HDV RNA was undetectable or below the lower limit of detection (LLOD). An intermediate virological response was characterized by an HDV RNA decline of 1 ≤ HDV RNA ≤ 2 Log. Virological breakthrough indicated an increase in HDV RNA of ≥1 Log after the nadir (lowest value of HDV-RNA).

### 2.2. HBV and HDV Serological Markers

HBsAg, antibodies to HBsAg (anti-HBs), HBeAg, and the corresponding antibody (anti-HBe) were analyzed in serum using a chemiluminescent microparticle assay with the Alinity instrument (microparticle enzyme assay, Abbott Diagnostics, Chicago, IL, USA). HDV IgG and IgM were detected using the DIAPRO assay, according to the manufacturer’s instructions (DIAPRO, Milan, Italy).

HBV core-related antigen (HBcrAg) levels in serum from HBV/HDV-positive individuals were quantified using a chemiluminescence enzyme immunoassay (CLEIA) on the fully automated Lumipulse G600II system (Fujirebio Europe, Gent, Belgium), according to the manufacturer’s instructions. Briefly, 200 µL of serum was incubated with 200 µL Fujirebio pre-treatment solution at 60 °C for 30 min before analysis, employing the Lumipulse G600II system. Results were expressed as Log U/mL. The upper limit of quantitation of the Lumipulse HBcrAg assay was 7 Log U/mL, while the lower limit of detection was 2 Log U/mL.

The levels of Immunoglobulin G antibody to hepatitis B core antigen (anti-HBc IgG) in serum were measured using the Lumipulse^®^ G Anti-HBc IgG-N assay (Fujirebio, Tokyo, Japan). Here, 200 µL of serum was analyzed using the Lumipulse G600II system. Results were reported as a cut-off index (COI).

### 2.3. HBV RNA and DNA

HBV RNA levels were analyzed using the HBV RNA Quantitative Fluorescence Diagnostic Kit (Sansure Biotech Inc., Changsha, China). First, the PCR Master Mix was prepared by combining 24.5 µL of HBV RNA PCR Mix, 4 µL of HBV RNA Enzyme Mix, and 1.5 µL of RT Enhancer per reaction. RNA was extracted from serum samples, negative and positive controls, and quantitative references using the recommended magnetic bead-based nucleic acid extraction kit S10010 (LLOD 50 copie/mL). After digestion, 30 µL of the prepared Master Mix and 20 µL of the extracted RNA were added to each PCR reaction tube. Thermal cycling parameters included: pre-denaturation and enzyme activation at 95 °C for 1 min; reverse transcription at 60 °C for 30 min; cDNA pre-denaturation at 95 °C for 1 min; 45 cycles of denaturation at 95 °C for 15 s; annealing/extension and fluorescence collection at 60 °C for 30 s; and a final cooling step at 25 °C for 10 s. Results were analyzed based on amplification curves and control thresholds to determine HBV RNA levels.

HBV DNA viral load was quantified using the COBAS 6800 System (Roche Applied Science, Basel, Switzerland), and HDV RNA was measured with the Bosphore HDV RNA kit v1.0 (LLOD 100 IU/mL) (Anthalia, Istanbul, Turkey).

### 2.4. HDV Full Genome Determination

Viral RNA was isolated from serum samples using a commercial kit (QIAmp Viral RNA Minikit, QIAGEN, Hilden, Germany). cDNA was synthesized using random primers and the SUPERSCRIPT III enzyme (ThermoFisher, Waltham, MA, USA), following the recommended protocol. Amplification conditions and cycling were modified from protocols previously described by Celik et al. [28]. Fragments A and B were amplified using TaqGold DNA polymerase (ThermoFisher), employing the same assay conditions: 1× buffer, 1.5 mM MgCl_2_, 0.2 mM dNTPs, and 0.5 mM of sense and antisense primers. Amplification conditions for fragment A were: 94 °C for 10 min; 35 cycles at 94 °C for 30 s, 65 °C for 1 min, 72 °C for 2 min; and a final extension at 72 °C for 10 min. For fragment B: 94 °C for 10 min; 35 cycles at 94 °C for 40 s; 65 °C for 90 s; 72 °C for 3 min; and a final extension 72 °C for 10 min.

Positive samples were sequenced using dideoxynucleoside method (Big Dye TM terminator by Applied Biosystem Inc., Foster City, CA, USA).

Sequences were assembled using the BioEdit tool [29] and alignments were performed with ClustalW. Sequences were submitted to GenBank (accession numbers: PQ514005 and PQ673658-PQ673680).

### 2.5. Phylogenetic and Genotyping Analysis

The dataset consisted of 184 reference full-length HDV sequences and 30 patient-derived BL HDV sequences. Genotypes 2–7 were each represented by eight reference sequences, while genotype 8 was represented by six reference sequences. For genotype 1, the dataset included 27 sequences from subtype 1a, 30 from subtype 1b, 23 from subtype 1c, 45 from subtype 1d, and 6 sequences from subtype 1e. Of the 30 patient-derived sequences, 6 were partial sequences of various lengths (HDV017_CL_T0_A: 795 nt, HDV018_BVV_T0_A: 846 nt, HDV019_MV_T0_A: 688 nt, HDV020_SD_T0_A: 675 nt, HDV021_IT_T0_A: 837 nt, HDV022_MM_T0_A: 556 nt), while 24 were complete sequences.

For our phylogenetic and subtyping analyses, we first aligned all 30 patient-derived sequences against our reference dataset using the MAFFT-E-INS-i algorithm from MAFFT v7.511. To determine our prior for population growth, we ran BEAST v2.7.3 under a coalescent Bayesian Skyline model, a strict clock parameter, and a GTR evolutionary model. The MCMC algorithm was run for a chain length of 100 million with a sampling frequency every 10,000. Using Tracer v1.7.1, we then implemented a Bayesian Skyline reconstruction, which suggested that a Birth and Death population growth prior should be considered. Subsequently, we performed a Bayesian tree inference using BEAST v2.7.3 under a GTR Gamma Site Model, a strict Clock Model, and a Birth and Death prior model. Again, the MCMC algorithm was run for a chain length of 100 million samples, while re-sampling every 10,000. The final tree based on the 10,000 samples was inferred using Tree Annotator v2.7.3, with a burn in percentage equal to 10%. Compared to additional analyses on divided datasets, the combined Bayesian phylogenetic tree with all 30 patient-derived sequences provided the best tree resolution and was further used for subtyping.

Genotyping was assessed when a patient sequence would cluster with an earlier diverged reference sequence with a posterior probability > 95%. This included consecutive earlier diverged reference sequences from the same subtype if the resulting probability was also >95%. For example, if nodes A, B, and C had posterior probabilities 0.7, 0.8, and 0.9, respectively, the final probability was equal to 1 − (1 − 0.7) × (1 − 0.8) × (1 − 0.9) = ~0.99. Trees were inferred using BEAST v2.7.3 and Tree Annotator v2.7.3, while tree visualization was implemented with Figtree v1.4.4 and Adobe Illustrator 2025.

### 2.6. HDAg Protein Analysis

HDV sequences encoding the HDAg isoforms S-HDAg (195 amino acids [aa]) and the L-HDAg (214 aa) were translated using the BioEdit tool.

Shannon entropy values were calculated for each position in HDAg protein sequences to assess amino acid conservation, with conserved residues defined as those exhibiting zero entropy. In general, a low Shannon entropy value indicates a higher conservation of amino acids at specific positions within the protein sequence.

The S-HDAg isoform is involved in viral RNA synthesis and modulation of host interactions and contains several regions essential for its functionality. Among these, the RNA binding domain (RBD, aa2-27, aa97-107, and aa136-146) is crucial for binding to the HDV ribonucleoprotein (RNP) complex. Additionally, the coiled-coil sequence (CCS, aa31-52) mediates the polymerization of HDAg, while the nuclear localization signal (NLS, aa68-88) is responsible for the translocation of the HDV RNP from the cytoplasm to the nucleus.

The L-HDAg isoform is required for viral packaging, but is also a transdominant inhibitor of HDV RNA synthesis. It has an extra 19 amino acids at its C-terminus containing the virus-assembly signal (VAS, aa196-214), which includes a nuclear export signal (NES, aa198-210) and a Carboxy-terminal CXXQ sequence (CXXQ motif) (aa211-214), both of which inhibit HDV RNA amplification and promote the assembly of progeny viruses [30,31]. The CXXQ motif is essential for farnesylation, anchoring L-HDAg to the endoplasmic reticulum membrane to interact with HBsAg for viral particle assembly [32,33].

Patients were categorized into two groups based on their virologic responses: v-responders and v-non-responders. The analysis encompassed baseline HDAg sequences. To assess the potential relationship between specific mutations, pattern mutations, and treatment outcomes, k-means clustering was used to classify patients according to the slope of their HDV RNA kinetics, derived from linear regression models fitted to their viremia data over time. Additionally, Fisher’s exact test was employed to examine the statistical significance of mutation distribution among the identified clusters.

The HDAg multiple sequence alignment was conducted with ClustalW. Shannon entropy analyses and clustering were performed using the R programming language [34].

### 2.7. HDV RNA Secondary Structure Analysis

Analyses of the HDV secondary structure and relative mutations of antigenome RNA, focusing on the amber/W region, were conducted to investigate the relationship between HDV RNA structural changes and clinical outcome. The host enzyme adenosine deaminase, acting on RNA 1 (ADAR1), is primarily responsible for editing the HDV RNA at the amber/W site, converting an adenosine (A) to inosine (I), which changes the stop codon to a tryptophan codon, resulting in the production of L-HDAg from the S-HDAg [35].

The RNA secondary structure around the amber/W site is crucial for efficient editing. With the focus on HDV genotype 1 sequences, several structural requirements necessary for the editing process were evaluated [36,37]. Specifically, these requirements included an unbranched rod-like structure; the presence of A-C mismatch pairs located among eight canonical Watson–Crick base pairs at the amber/W site; and the observation that the structure of the amber/W site contains base-paired segments often disrupted by bulges, mismatches, and small internal loops necessary for the interaction with the double-stranded RNA binding motifs (DRBMs) of ADAR1 [36,37,38].

HDV antigenome RNA was established from full-length HDV genome consensus sequences using the BioEdit tool.

Prediction of HDV antigenome RNA secondary structures was achieved using the RNAfold WebServer (http://rna.tbi.univie.ac.at//cgi-bin/RNAWebSuite/RNAfold.cgi, accessed on 23 September 2024). The secondary structures were selected based on the minimum free energy (MFE) and their unbranched rod-like shape [17].

### 2.8. Statistical Analysis

Continuous variables were summarized as medians and interquartile ranges (IQRs), while categorical variables were expressed as frequencies and percentages.

To compare the baseline and follow-up characteristics between v-responders and v-non-responders, as well as the Shannon entropy values from HDAg protein sequences, the Mann–Whitney U test was employed to assess continuous variables. Longitudinal analysis of continuous variables was assessed using the Friedman test for repeated measures, followed by Dunn’s post hoc test for pairwise comparisons when significant. Finally, the Fisher’s exact test was used to evaluate any association between categorical variables and specific outcomes. A *p*-value < 0.05 was considered statistically significant. All statistical analyses were conducted using GraphPad Prism 9 software (GraphPad software, Inc., La Jolla, CA, USA) and RStudio 2024.09.0+375 (RStudio, Boston, MA, USA).

## 3. Results

### 3.1. Baseline Characteristics of Study Population

Of 30 patients with chronic HBV/HDV infection enrolled in our study, 6 received at least 24 weeks of BLV treatment, while 24 received 48 weeks.

Baseline characteristics are presented in Table 1 below.

The median age of the study participants was 49 years, and half were male. The median body mass index (BMI) was 24 (22.3–28.5). Cirrhosis was present in 70% of patients, while 73.3% had previously undergone interferon-based therapy. All patients received concomitant HBV NUC therapy. At baseline, median ALT and AST levels were 82.5 (54.5–103.25) U/L and 71.0 (62–89) U/L, respectively. In addition, median levels of albumin, bile acids, total bilirubin, and platelet count were 4.2 (4.0–4.4) g/dL, 9.45 (5.5–16.6) μmol/L, 0.81 (0.69–1.54) mg/dL, and 113.5 (81.8–161.5) × 10^3^/μL, respectively. Regarding virological characteristics, the median HDV RNA level was 4.6 (3.8–5.7) Log copies/mL (cp/mL) with 60.7% of patients having detectable HBV DNA. Finally, the median values of HBsAg, HBcrAg, and anti-HBc IgG were 3.9 (3.6–4.2) Log IU/mL, 3.3 (2.6–4.1) Log U/mL, and 105 (37.7–298.1) COI, respectively.

Patients who achieved a virological response were defined as “v-responders”, while patients who did not achieve a virological response were defined as “v-non-responders”.

### 3.2. Virological and Biochemical Response

**Figure 1:**
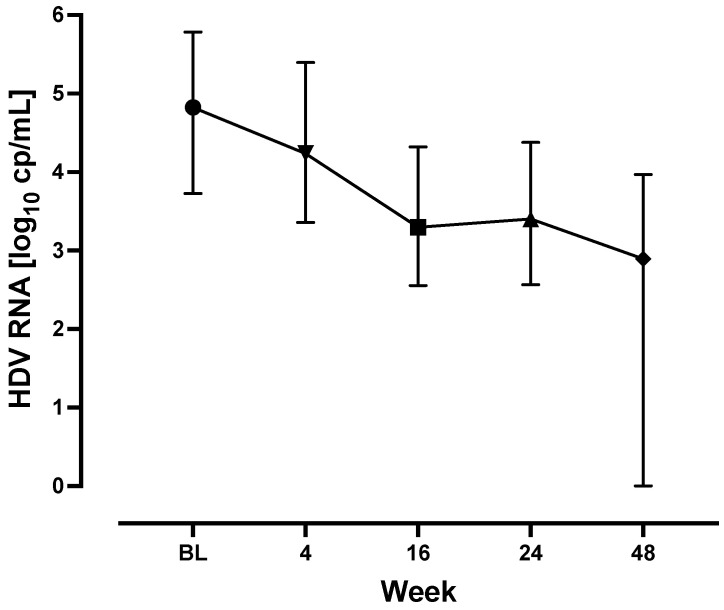
Virological kinetics. Median HDV RNA levels are shown with the interquartile range at different study time points for the entire patient cohort. The whiskers represent the interquartile range. Baseline, BL.

When considering the entire patient cohort, median HDV RNA levels decreased significantly from 4.9 (3.9–5.8) Log cp/mL at BL to 2.9 (0.0–3.8) Log cp/mL at TW48 (*p* < 0.001) (Figure 1, Table 2). Median declines in HDV RNA were 1.4 (0.6–2.3), 1.3 (0.2–2.1), and 2.0 (0.1–3.2) Log cp/mL at weeks 16, 24, and 48, respectively, when compared with BL.

Undetectable HDV RNA was achieved in 5/30 (33.3%) patients at TW24 compared with 7/24 (58.3%) at TW48 (Figure S1A). At TW16, a significant reduction in HDV RNA was observed in 19/30 (63.3%) of patients (*p* < 0.0001). After this time point, two distinct patterns emerged among patients. Sixteen individuals maintained a sustained decline in viral load, achieving a virological response at TW24 or TW48 (Figure S1A). Conversely, two patients showed an increase in viral load indicative of a virological breakthrough (Figure S1C); therefore, they were considered within the group of v-non-responders (also see Figure S1B). Consequently, for our analyses, 16 patients were defined as v-responders, while 14 were v-non-responders during the observation period.

HDV RNA at BL did not statistically differ between v-responders (5.0 [4.1–5.7] Log cp/mL) and v-non-responders (4.3 [3.9–5.9] Log cp/mL) and did not correlate with clinical outcome (*p* = 0.45) (Table S1 and Table S2).

In general, HBV DNA levels did not vary throughout the observation period, with 29/30 (96.6%) of patients exhibiting undetectable or detectable values < 10 IU/mL. In fact, HBV RNA values were undetectable at BL, TW24, and TW48 in samples from all 30 patients.

Median HBsAg values were comparable throughout the observation period (Table S1, Table S2 and Table 3), with no significant decrease in v-responders (*p* = 0.98) or v-non-responders (*p* = 0.12). Similarly, median HBcrAg values did not show statistically significant differences overall (Table 3). Moreover, HBcrAg values were not statistically different between v-responders and v-non-responders at any time point (Table S1 and Table S2): At BL, the values were 3.2 (2.7–4.1) Log U/mL and 3.5 (2.6–4.0) Log U/mL; at TW24, they were 3.2 (2.4–3.9) Log U/mL and 3.6 (2.9–4.2) Log U/mL; and at TW48, they were 3.4 (2.6–4.2) Log U/mL and 3.3 (3.0–4.0) Log U/mL, respectively.

**Figure 2:**
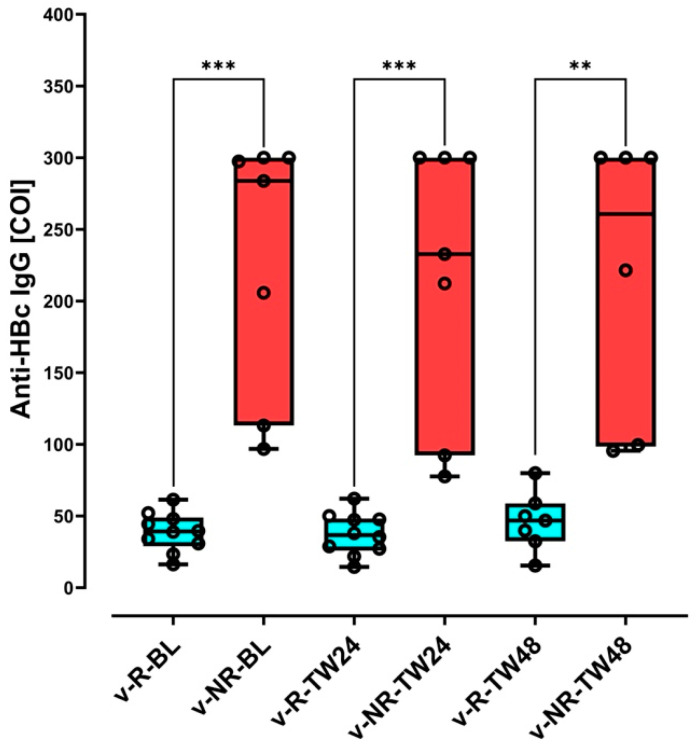
Box plot of immunoglobulin G antibody to hepatitis B core antigen (Anti-HBc IgG) values in v-responders (individuals who achieved a virological response) and v-non-responders (individuals who did not achieve a virological response). The box plot depicts anti-HBc IgG values for patients at baseline (BL), 24 weeks (TW24), and 48 (TW48) weeks of BLV treatment. V-responders are represented in blue. V-non-responders are represented in red. The whiskers indicate minimum and maximum values. v-R-BL, v-responders at baseline; v-NR-BL, v-non-responders at BL; v-R-TW24, v-responders at TW24; v-NR-TW24, v-non-responders at TW24; v-R-TW48, v-responders at TW48; v-NR-TW48, v-non-responders at TW48; ***, *p* = 0.0001; **, *p* = 0.0012.

Interestingly, median anti-HBc IgG values were lower in v-responders when compared to v-non-responders (Figure 2, Table S1 and Table S2). Specifically, v-responders had a median value of 39.3 (31.6–47.1) COI at BL versus 244.7 (127.0–299.4) COI for v-non-responders. At TW24, median values for v-responders and v-non-responders were 36.7 (27.6–47.6) COI and 222.5 (105.4–300.0) COI, respectively. Finally, at TW48, median anti-HBc IgG values were 49.9 (39.9–79.9) and 260.7 (130.0–300.0) COI for v-responders and v-non-responders, respectively. Differences were statistically significant between v-responders and v-non-responders (Figure 2) at BL (*p* = 0.0001), TW24 (*p* = 0.0001), and TW48 (*p* = 0.0076). However, median anti-HBc IgG values within each of the v-responders (*p* = 0.77) and v-non-responders (*p* = 0.44) did not differ statistically during the treatment observation period.

Median ALT levels declined from 82.5 (54.5–103.3) U/L at BL to 32.5 (22.3–42.8) U/L at TW24, and further to 30.0 (20.3–45.3) U/L at TW48.

A similar trend was observed for AST levels, which decreased from a median value of 71.0 (62.0–89.0) U/L at BL to 35.5 (28.0–42.0) U/L at TW24, and to 33.0 (28.0–49.5) U/L at TW48. Overall, the combined normalization of ALT and AST was achieved in 55.0% of patients, regardless of virological response, after 24 weeks of treatment. Furthermore, this normalization remained largely unchanged even at 48 weeks post-treatment (54.5%).

In general, a biochemical response, defined as ALT normalization (ALT < 40 U/L), was achieved in 66.7% of the cohort at TW24 and in 63.6% at TW48. Meanwhile, a combined response (both biochemical and virological responses) for the entire cohort was achieved in 46.6% of patients at TW24 and in 45.8% of patients at TW48.

When considering the serological markers, i.e., albumin, total bilirubin, and platelet count, their median values remained unchanged during treatment (see Table 1 and Table 3). In contrast, median bile acid values increased from 9.5 (5.5–16.7) μmol/L at BL to 35.8 (18.1–60.6) μmol/L at TW24 (*p* = 0.016) and to 24.5 (17.8–32.3) μmol/L at TW48 (*p* = 0.007).

### 3.3. Genotype and Phylogenetic Analysis

**Figure 3:**
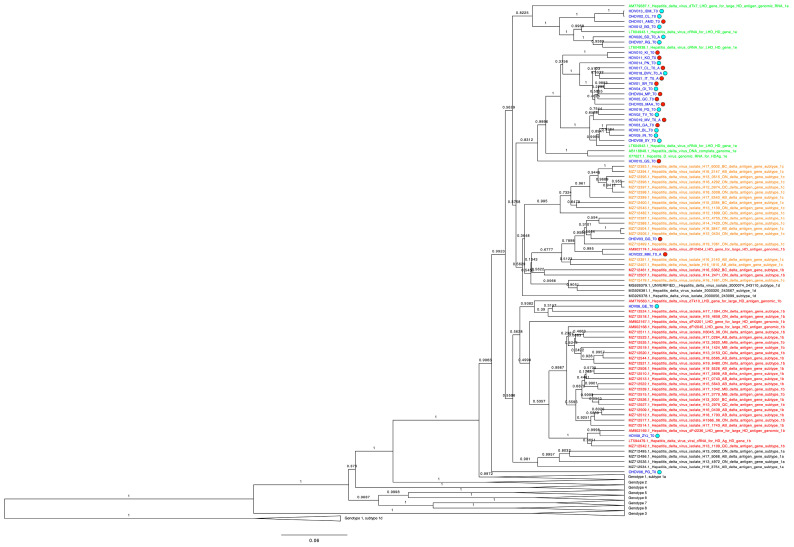
Bayesian phylogenetic tree. The tree was constructed using fragment A, which was available for all 30 patients (see Materials and Methods, Section 2.5). Patient-derived BL sequences are colored blue (blue circles represent virological responders and red circles represent virological non-responders), while reference sequences are colored red (subtype 1b), orange (subtype 1c), and green (subtype 1e). As there were no patient sequences clustering with lineages for genotypes 2 through 8, as well as genotype 1 subtypes 1d and subtype 1a (partially), as indicated by a high posterior probability, they were collapsed for convenience. Sequences were aligned using MAFFT v7.511, the phylogenetic tree was inferred using BEAST v2.7.3 and Tree Annotator v2.7.3, and tree visualization was implemented with Figtree v1.4.4 and Adobe Illustrator 2025.

To subtype HDV sequences from 30 HBV/HDV coinfected patients, a Bayesian tree was inferred using a Birth and Death prior model employing a strict clock and a GTR site model. Based on the resulting tree (Figure 3 and Figure S2), 25 sequences clustered with subtype 1e reference sequences (HDV012_BG_T0, HDV020_SD_T0_A, OHDV07_RG_T0, HDV013_IDM_T0, OHDV02_CL_T0, OHDV01_AMD_T0, OHDV05_MAA_T0, HDV05_GC_T0, OHDV04_MP_T0, HDV04_OI_T0, HDV01_SR_T0, HDV021_IT_T0_A, HDV018_BVV_T0_A, HDV017_CL_T0_A, HDV014_PN_T0, OHDV08_SY_T0, HDV09_IN_T0, HDV07_BL_T0, HDV03_GA_T0, HDV019_MV_T0_A, HDV02_TV_T0, HDV016_PG_T0, HDV011_KO_T0, HDV010_KI_T0, HDV015_GS_T0), 2 sequences clustered with subtype 1b (HDV08_ZYJ_T0, HDV06_GE_T0), and 1 sequence clustered with subtype 1c (OHDV03_GG_T0). Sequence HDV022_MM_T0_A appeared to cluster with subtype 1c, although the specific lineage was not clearly defined, due to having multiple reference sequences from subtypes 1b, 1c and 1d. Finally, sequence OHDV06_PG_T0 had an undefined genotype subtype.

Specifically, of the 16 v-responders, 13 were classified as subtype 1e, 2 as subtype 1b, and 1 as undefined. Meanwhile, among the 14 v-non-responders, 12 were classified as subtype 1e and 2 as subtype 1c.

As shown in Figure 3, no specific viral strains were associated with therapy failure, and subtype 1e was predominant across the cohort. Additionally, no distinct clusters correlated with treatment outcome, as sequences from v-responders and v-non-responders were intermixed.

### 3.4. HDAg Protein Analysis

Delta antigen (HDAg) protein sequences were analyzed for the 24 patients who achieved at least 48 weeks of treatment. Sequences from 14 v-responders and 10 v-non-responders (including 1 patient with virological breakthrough) were compared to the reference sequence, WGH71036.1 (large delta antigen (Hepatitis delta virus)). Comparisons of amino acid polymorphisms in the functional domains of HDAg protein among v-responder and v-non-responder patients are shown in Figure S3.

**Figure 4:**
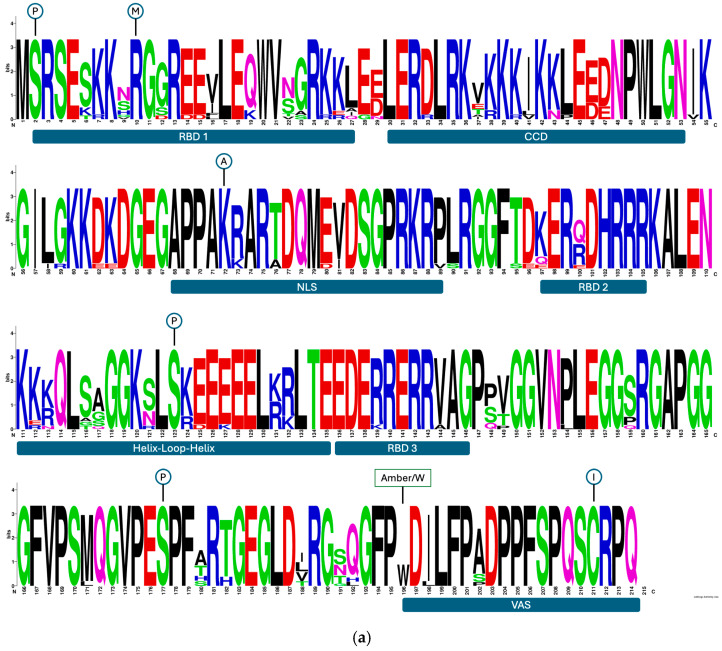

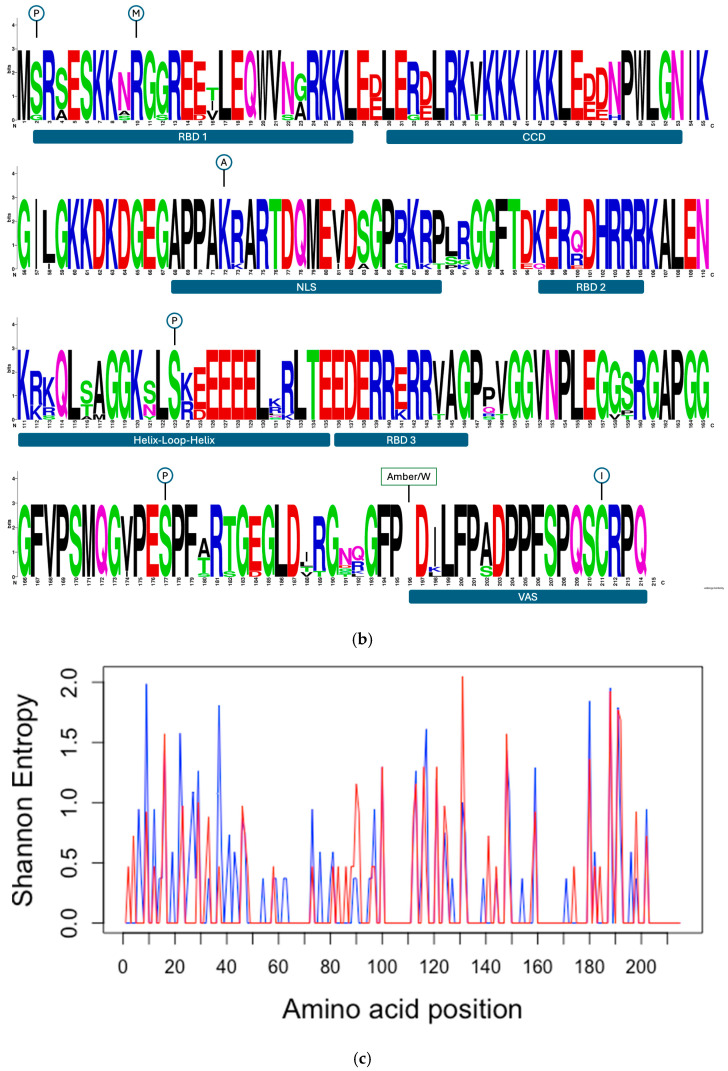
(**a**) Representation of baseline hepatitis delta antigen (HDAg) protein sequences derived from 14 HBV/HDV-positive patients who achieved virological response (v-responders) and (**b**) from those (n = 10) who did not achieve virological response (v-non-responders). Post-transcriptional modification (PTM) sites are indicated by circles, while functional domains are represented by boxes. Sequence logos were generated using WebLogo 3. (**c**) The line plot illustrates the Shannon entropy profiles for two groups of sequences derived from the HDAg protein: v-responders (indicated in blue) and v-non-responders (indicated in red). The *x*-axis corresponds to the amino acid positions along the HDAg sequence, while the *y*-axis represents the corresponding Shannon entropy values. P, phosphorylation; M, methylation; A, acetylation; I, isoprenylation; RBD, RNA-binding domains; CCD, coiled-coil domain; NLS, nuclear localization sequence; VAS, virus-assembly signal.

The conservation of amino acids in the HDAg protein was analyzed across specific functional domains (RBD1, RBD2, RBD3, CCD, NLS, NES, VAS, and CXXQ motif), and a comparison was made for sequences derived from v-responders and v-non-responders, respectively, using Shannon entropy (Figure 4c).

All residues at the post-translational modification (PTM) sites (see Figure 4a,b) were conserved, except for a single substitution: a serine-to-glycine change at position 2 in a v-non-responder patient with subtype 1e. This substitution was previously reported in other studies [39].

The percentage of HDAg amino acid conservation (Shannon entropy = 0) was lower in v-responders (69.3%) than v-non-responders (75.8%). In fact, the Shannon entropy of full-length BL HDAg protein sequences from v-responders was higher than from v-non-responders (mean ± SD: 0.24 ± 0.45 for v-responders and 0.20 ± 0.41 for v-non-responders), although not significant (*p* = 0.226).

Assessment of specific functional domains revealed that the RBD1 domain showed significantly lower conservation in v-responders (50.0%) compared to v-non-responders (69.2%). Indeed, the RBD1 domain in v-responders exhibited greater variability than in v-non-responders (mean ± SD: 0.44 ± 0.58 for v-responders and 0.23 ± 0.41 for v-non-responders; *p* = 0.161, Mann–Whitney test). Specifically, in the RBD1 domain, amino acid position N22 showed high variability in v-responders (Shannon entropy: 1.57) compared to v-non-responders (Shannon entropy: 0.47) (Table S3). Additionally, certain substitutions were observed exclusively in v-responders: N22V (subtype 1e) and N22T (undefined subtype). Although amino acid positions N9 (Shannon entropy: 1.99 for v-responders and 0.92 for v-non-responders) and V16 (Shannon entropy: 1.43 for v-responders and 1.57 for v-non-responders) were highly variable in both groups, the amino acid changes N9H (subtype 1e), N9G (undefined subtype), and N9V (subtype 1b) were present only in v-responders, while V16T (subtype 1e) was only found in v-non-responders. Finally, the G23A polymorphism was more prevalent in v-non-responders than v-responders, with Shannon entropy of 0.97 and 0.73, respectively.

The CCD domain followed a similar trend, with 59.1% conservation in v-responders and 72.7% in v-non-responders (Shannon entropy mean ± SD: 0.29 ± 0.45 for v-responders and 0.18 ± 0.32 for v-non-responders; *p* = 0.417). Notably, the D33E polymorphism was only detected in v-non-responders (3/10 patients). In addition, amino acid V37 exhibited higher variability in v-responders (42.9%) than in v-non-responders (10%), which aligned with the Shannon entropy values of 1.81 and 0.47, respectively. Specifically, the V37E polymorphism was only detected in v-responders (subtype 1b). Furthermore, the K43N polymorphism was only detected in v-responders, while the same position was highly conserved in v-non-responders (Shannon entropy: 0).

In contrast to the above domains, the NLS domain exhibited higher conservation in v-responders (81.0%) compared to v-non-responders (76.2%). The T76A polymorphism was only detected in v-responders (n = 2 subtype 1e).

In the v-non-responders, the V16T and G23A polymorphisms were detected in the same set of patients, specifically four individuals classified with subtype 1e. Among these four patients, two also had an S4A polymorphism and presented with HDV RNA levels of 5.87 and 3.46 Log cp/mL at BL and 5.76 and 3.36 Log cp/mL at TW48, respectively. The D33E polymorphism was also detected in one of the four patients, with HDV RNA levels of 3.29 Log cp/mL at BL and 3.23 Log cp/mL at TW48. Notably, a combination of four substitutions (S2G (IN PTM), V16T, G23A, and D33E) was detected in one of these patients with HDV RNA levels of 3.91 Log cp/mL at BL and 3.36 Log cp/mL at TW48.

In other functional domains, the same conservations were observed for both v-responders and v-non-responders: RBD2 (81.8%), RBD3 (81.8%), NES (84.6%), and VAS (88.9%). In these domains, the detected polymorphisms were the same between the two groups, except for the K97E polymorphism in RBD2 and the polymorphisms R139K and V144A in RBD3; these were detected exclusively in v-responders (subtype 1e). Finally, the CXXQ motif was highly conserved in both groups (100%); in fact, the sequence was the same in all patients: CRPQ, as reported in other studies.

Overall, these results revealed multiple polymorphisms in the HDAg protein, with shared variants observed in both v-responders and v-non-responders, such as N9S, Q100R, and E46D. Unique polymorphisms, including N22S and T76A in v-responders and D33E, V16T, and E141K in v-non-responders, were noted, but occurred at low frequencies. The combination analysis demonstrated no recurrent patterns of baseline polymorphisms within groups, and none appeared to impact clinical response to treatment.

Within the v-responder group, two distinct clusters based on viremia slopes were identified using k-means clustering. Cluster 1 indicated a rapid decline in viremia, whereas Cluster 0 reflected a moderate decline. V-responders in Cluster 1 exhibited frequent polymorphisms including D47E and E46D, while those in Cluster 0 included polymorphisms G12S, V16F, and A202S/P. Similarly, v-non-responders were stratified into Cluster 0, characterized by stable or increasing viremia, and Cluster 1, which displayed a slight decline. In Cluster 1, the most frequently detected polymorphisms were V81I and I198L, whereas D33E and D47E were in Cluster 0.

No significant differences in polymorphism distribution across clusters for both v-responders and v-non-responders were found (*p* = 0.4). The sporadically detected polymorphisms observed did not appear to correlate with treatment efficacy.

### 3.5. Secondary Structure Analysis

HDV antigenome RNA secondary structures and their respective mutations were analyzed using BL patient-derived full-length genome sequences for 24 patients receiving 48 weeks of treatment (Figure S4). Fourteen patients were v-responders and 10 were v-non-responders (including 1 patient with virological breakthrough).

Analysis of the HDV editing site focused on structural features associated with patient response to therapy, including parameters such as the presence of A-C mismatches, the number of DRBMs within the 25 nucleotides 3′-downstream of the editing site, and length, defined as the number of consecutive nucleotides base-pairing for each DRBM (e.g., 4-8-6) (Table 4).

Among the v-responders, an A-C mismatch in the RNA structure was observed in 12 patients (86%), but not in 2 patients (14%). Baseline HDV RNA levels for the latter two patients were 5.04 Log cp/mL and 2 Log cp/mL, which decreased by 2.7 Log cp/mL and 2 Log cp/mL, respectively, at TW48. In comparison, among the v-non-responders, nine patients (90%) had an A-C mismatch, while one patient (10%) did not. The BL HDV RNA value for the one patient without an A-C mismatch was 3.1 Log cp/mL at BL, which only decreased by 0.1 Log cp/mL at TW48.

Notably, the three patients lacking the A-C mismatch (two v-responders and one v-non-responders) at the editing site had a guanine (G) instead of the adenosine (A). This substitution results in a G-C pair, which is not a substrate for ADAR1, since the enzyme specifically targets adenosines within double-stranded RNA regions. Consequently, the absence of the target adenosine prevents the editing event necessary for producing L-HDAg [37]. Where present, the A-C mismatch pair was detected in the middle of eight canonical Watson–Crick base pairs for all BL patient-derived sequences.

Regarding the number of DRBMs, 12 v-responders (86%) had 3 DRBMs, and 2 v-responders (14%) had 4 DRBMs. Among the v-non-responders, nine patients (90%) had three DRBMs, and one patient (10%) had four DRBMs. Finally, the structural length (4-8-6) was predominant across the groups. The above findings suggest that differences were minor between the two groups. Furthermore, when considering additional clinical parameters such as the rate of HDV RNA decline, no correlation was observed between these parameters and the structural features of the HDV amber/W site in either v-responders or v-non-responders (*p* > 0.9999). The results suggest that, except for the absence of the A-C mismatch, the RNA structural requirements necessary for editing were present in all patients. For this reason, other characteristics were also evaluated, such as the region between 63 and 100 nucleotides downstream on the 3′ end, known to be rich in base pairings that facilitate the editing process, as well as the upstream region adjacent to the editing site (30 nucleotides before the editing site) [36,37].

V-responders displayed a higher frequency of structures with no internal loops (6/14, 43%) compared to v-non-responders (1/10, 10%), a difference that was statistically significant (*p* = 0.033). Patients with one internal loop were observed in both groups with no significant difference (v-responders: 7/14, 50%; v-non-responders: 3/10, 30%; *p* > 0.05). In contrast, two internal loops were significantly more common in v-non-responders (6/10, 60%) compared to v-responders (1/14, 7%) (*p* = 0.0088).

No significant differences in internal loop distribution were observed in regions adjacent to the editing site, such as the upstream region (30 nucleotides before the editing site) between v-responders and v-non-responders. However, these areas appeared to exhibit less structural variability overall, suggesting a higher level of conservation compared to the region 64–100 nucleotides downstream.

## 4. Discussion

Bulevirtide monotherapy has been approved in the European Union as a treatment for patients with chronic HDV infection. In this study, we investigated potential predictive factors for virologic (and combined) response in HBV/HDV-coinfected patients treated with bulevirtide at 2 mg/day for up to 48 weeks. The following biomarkers were evaluated: HDV RNA, HBV RNA, HBcrAg, and Anti-HBc IgG. We also analyzed the full-length HDV genome to assess whether specific baseline HDV polymorphisms correlated with treatment outcomes. Despite the wide range in baseline HDV RNA levels (100 to 28,652,175 cp/mL), no significant difference in response was observed between v-responders and v-non-responders (*p* > 0.05).

All patients were infected with HDV genotype 1, with subtype 1e being the predominant subtype (n = 25, 83%). Amongst our study population, the genotype subtype did not appear to impact the response to bulevirtide treatment, with the caveat of a limited sample size. This result is in agreement with previously reported findings [40].

HBsAg levels showed no significant decrease during the observation period (TW48), nor a difference between v-responders and v-non-responders, consistent with other studies. This may be related to BLV not interfering with HBV expression at the cccDNA level or in the integrated form. Surrogate marker levels for transcriptional activity (HBV RNA) also provided no insight, since patients had HBV RNA <100 cp/mL at baseline and at week 48. The sensitivity threshold (50 cp/mL) of our test may possibly explain the differences from the results of Hollnberger et al.’s [40], who detected HBV RNA at 20 cp/mL. Our findings suggest that serum may not be optimal for HBV RNA detection. In fact, as described by Degasperi [41], only 8% of patients exhibited detectable HBV RNA in serum compared to 78% in liver biopsies, indicating active transcription from cccDNA in the liver.

Another biomarker, HBcrAg, which correlates with cccDNA transcriptional activity, similarly lacked predictive value. Median baseline values were 3.3 (2.6–4.1) Log U/mL, with no significant changes between v-responders and v-non-responders at any time points. This aligns with the lack of transcriptional influence by BLV; however, other studies [42] have noted significant declines in HBcrAg at TW24. The transient decline may be due to the small sample size in our study and Sandmann’s, as values were similar, making it difficult to establish a predictive baseline or a cut-off value for BLV response.

Baseline anti-HBc IgG values exhibited the greatest predictive potential. Study patients with lower baseline anti-HBc IgG attained virological responses at TW24 and TW48 (*p* = 0.0001 at baseline; *p* = 0.0001 at TW24; *p* = 0.0076 at TW48). Studies have shown that high anti-HBc IgG levels correlate with increased inflammation [43,44,45,46,47]. Similarly to HBV antiviral treatment, anti-HBc IgG was predictive of BLV response in HBV/HDV-coinfected patients. Lower baseline values (39.3 [31.6–47.1] COI) correlated with virological response at TW24 and TW48, and while higher values (244.7 [127.0–299.4] COI) were observed in v-non-responders (Figure 2), no statistical variation was determined during the observation period.

The majority of our patient cohort belonged to subtype 1e, comprising 13 v-responders and 12 v-non-responders. Among the five non-1e patients, two with subtype 1b achieved a virological response, while two with subtype 1c and one with an undefined subtype did not respond. The subtype may influence the response to the drug; however, due to the limited number of cases analyzed, we are unable to draw any definitive conclusions.

From our baseline analysis of the HDAg protein, no exclusive and recurrent polymorphism patterns were identified in either response group. The amino acid conservation rate was similar between v-responders (69.3%) and v-non-responders (75.8%), and the observed polymorphisms appeared randomly distributed without association with known functionally relevant protein regions concerning treatment response. Additionally, we found that the L-HDAg CXXQ motif was highly conserved among all examined patient-derived baseline sequences; specifically, the CRPQ sequence was identical across both v-responders and v-non-responders. Since this crucial protein motif was conserved, its role in viral assembly and completion of the HDV life cycle should not be impaired [32,33]. Therefore, an impact on clinical response to BLV treatment is unlikely.

The majority of HDAg variants reported by Hollnberger et al. [40] were not detected in our analyses, except for S2G (in 1 v-non-responder), E125D (2 v-non-responders, 1 v-responder), E184D (1 v-non-responder), K25R (1 v-responder), I54V (1 v-responder), and D62E (1 v-responder), possibly due to the diversity of subtypes. Additionally, k-means clustering identified distinct substitution patterns between v-responders and v-non-responders based on viremia slopes, but no significant correlation between these substitutions and HDV viremia kinetics was found, suggesting that these amino acid substitutions did not impact treatment response. Therefore, our analysis of the HDAg protein underscores the sporadic nature of HDAg substitutions, indicating that baseline polymorphisms did not correlate with virologic response to BLV treatment, which is consistent with literature findings [40].

ADAR1 is essential for the RNA editing of HDV at the amber/W site, facilitating the production of L-HDAg necessary for viral packaging. The HDV RNA secondary structure around the editing site is critical for efficient editing, which in turn regulates the HDV replication cycle. Therefore, HDV antigenome RNA secondary structures were derived from baseline samples for the 24 patients (14 v-responders and 10 v-non-responders) treated with BLV for 48 weeks, with a focus on the region surrounding the amber/W site. Except for the absence of the A-C mismatch identified in three patients (two v-responders and one v-non-responder), RNA structural requirements necessary for editing the HDV antigenome RNA [38] were present across the cohort, and the differences were minor between v-responders and v-non-responders. Our findings suggested that a fully base-paired stem without internal loops in the 63–100 nt region 3′ of the editing site was associated with a better virological response (*p* = 0.033), possibly by stabilizing the RNA structure and enhancing the efficiency of ADAR1-mediated editing [48]. Conversely, the presence of two internal loops in this region might have destabilized the RNA structure, impairing editing efficiency and contributing to poorer therapeutic outcomes (*p* = 0.0088) [36,37]. Notably, increased RNA editing has been shown to correlate with potent inhibition of HDV RNA replication, indicating that increased L-HDAg production is the primary mechanism for S-HDAg inhibition [49]. In contrast, reduced editing efficiency might compromise the production of L-HDAg, reducing S-HDAg inhibition and favoring viral persistence. Consequently, S-HDAg molecules can be produced and subsequently transmitted to hepatocyte progeny. In fact, according to the Giersch’s study [50], HDV can persist during liver regeneration by transferring HDV RNA to dividing hepatocytes, even in the absence of concurrent HBV infection. Furthermore, this might explain why the mathematical modeling study by Shekhtman [51] suggested the existence of two populations of HDV-infected cells. This model helps account for why the expected monophase response with the complete BLV-mediated viral entry blockage did not match the experimentally observed biphasic pattern, suggestive of two cellular populations with different HDV clearance rates: one with rapid clearance and one with slow clearance. This also explains why, at week 144, only 50% of the patients had detectable HDV RNA levels with a 10 mg dose of BLV [27]. Therefore, HDV’s capacity for persistence may explain the difficulties in achieving HDV clearance among patients with chronic HBV/HDV coinfection receiving BLV treatment.

This study has some limitations. For instance, we did not analyze the pre-S1 region involved in the interaction with the Na^+^-taurocholate co-transporting polypeptide (NTCP). However, this was not feasible, since patients receiving NUC therapy exhibited HBV DNA viremia ≤ 10 IU/mL, and amplification of this region yielded no results. Nonetheless, studies that have investigated this region have reported the absence of specific mutations in non-responders compared to responders [40]. Additionally, we did not assess for polymorphisms in the NTCP receptor-coding region. However, the small sample size and presence of patients in our study from the Balkan countries (Romania and Moldova) likely would not have provided conclusive results, as suggested by a recent study by Toniutto et al. [52]. Toniutto et al. found that the genetic rs17556915 polymorphism TT/CC was predictive of higher baseline values while the TT/CT polymorphism was not; however, definitive information on the response to BLV was not described. Furthermore, polymorphisms may be associated with the ethnicity of the patients [53]. Analyzing a larger patient cohort and assessing the distribution of these polymorphisms in the general population may provide more robust insights into potential associations between NTCP polymorphisms and the response to BLV.

## 5. Conclusions

Our study highlights the complex interplay of various factors influencing the virological response to BLV treatment in patients with chronic HBV/HDV co-infection. Notably, lower baseline anti-HBc IgG levels emerged as the most significant predictor of virological response, underscoring the potential role of hepatic inflammation in treatment efficacy. Additionally, we identified unique HDAg polymorphisms in both v-responders and v-non-responders; however, the limited number of observed cases prevented us from drawing any definitive conclusions.

To our knowledge, this is the first study to analyze the secondary structure of HDV RNA in patients receiving BLV treatment. Our preliminary results highlighted a higher efficiency of editing in patients who achieved a virological response to BLV treatment, suggesting the need for further investigation in this area. The most significant factor associated with the response to BLV was identified as anti-HBc IgG, indicating that the inflammatory state of the liver may serve as a discriminative element for the success of BLV therapy.

## Figures and Tables

**Table 1:** Baseline clinical and virological characteristics.

Parameter	N = 30
Age at recruitment, years	49 (45–59)
Male sex	14 (46.7)
Body mass index	24 (22.3–28.5)
Cirrhosis	21 (70.0)
Previous interferon therapy	22 (73.3)
Concomitant NUC therapy	30 (100)
ALT, U/L	82.5 (54.5–103.3)
AST, U/L	71.0 (62–89)
Albumin, g/dL	4.2 (4.0–4.4)
Bile acids, μmol/L	9.45 (5.5–16.6)
Total bilirubin, mg/dL	0.81 (0.7–1.5)
Platelet count, ×10^3^/μL	113.5 (81.8–161.5)
HDV RNA, Log cp/mL	4.9 (3.9–5.8)
HBV DNA detectable *	17 (56.7)
HBsAg, Log IU/mL	3.9 (3.6–4.2)
HBcrAg, Log U/mL	3.3 (2.6–4.1)
Anti-HBc IgG, COI	(39.4–225.2)

* HBV DNA ≥ 10 IU/mL. Parameter values are either expressed as numbers (%) or medians (IQR1–IQR3). IQR, interquartile range; NUC, nucleos(t)ide-analogue therapy; ALT, alanine aminotransferase; AST, aspartate aminotransferase; HDV, hepatitis delta virus; HBV, hepatitis B virus; HBsAg, hepatitis B surface antigen; HBcrAg, hepatitis B core-related antigen; anti-HBc IgG, immunoglobulin G antibody to hepatitis B core antigen; COI, cut-off index.

**Table 2:** Statistical analysis of kinetic median HDV RNA levels across different time points in the entire study cohort.

	BL	TW4	TW16	TW24	TW48
BL					
TW4	ns *				
TW16	*p* < 0.0001	*p* = 0.006			
TW24	*p* = 0.0013	ns	ns		
TW48	*p* < 0.0001	*p* = 0.0013	ns	ns	

* ns = not significant. Significance *p* < 0.05.

**Table 3:** Characteristics of biochemical and virological variables during bulevirtide treatment.

Parameters	TW4	TW16	TW24	TW48
ALT, U/L	67.0 (46.0–87.5)	39.0 (32.2–54.7)	32.0 (22.0–42.0)	30.0 (20.3–45.3)
AST, U/L	50.5 (44–67.8)	40.0 (32.0–50.7)	37.0 (28.0–42.0)	33.0 (28.0–50.0)
Albumin, g/dL	4.1 (3.9–4.5)	4.2 (3.9–4.5)	4.3 (4.1–4.4)	4.3 (4.1–4.5)
Bile acids, μmol/L	27.1 (15.6–45.9)	26.1 (18.1–44.5)	35.8 (18.0–60.6)	24.5 (17.8–32.3)
Total bilirubin, mg/dL	0.78 (0.7–1.3)	0.84 (0.7–1.2)	0.82 (0.7–1.3)	0.80 (0.6–1.3)
Platelet count, ×10^3^/μL	118 (91.0–177.5)	120 (89.5–190.2)	129 (97.0–189.0)	128 (97.0–188.0)
HDV RNA, Log cp/mL	4.2 (3.5–5.3)	3.3 (2.6–4.2)	3.4 (2.7–4.4)	2.9 (0.0–3.8)
HBV DNA detectable *	17 (56.7)	11 (36.7)	11 (36.7)	9 (37.5)
HBsAg, Log IU/mL	3.9 (3.5–4.2)	4.0 (3.5–4.2)	3.9 (3.4–4.2)	4.0 (3.4–4.2)
HBcrAg, Log U/mL	3.4 (2.6–4.2)	3.3 (2.8–4.0)	3.4 (2.5–3.9)	3.3 (2.8–4.1)
Anti-HBc IgG, COI			56.3 (37.4–217.3)	95.5 (48.4–225.6)
Number of responders **	3/30 (10.0)	11/30 (36.6)	10/30 (33.3)	14/24 (58.3)

* HBV DNA ≥ 10 IU/mL. ** Patients with a decrease in HDV RNA ≥ 2 Log cp/mL. Parameter values are either expressed as numbers (%) or medians (IQR1-IQR3). Data provided at TW4, TW16, and TW24 correspond to 30 patients, while data at TW48 correspond to 24 patients. NUC, nucleos(t)ide-analogue therapy; ALT, alanine aminotransferase; AST, aspartate aminotransferase; HDV, hepatitis delta virus; HBV, hepatitis B virus; HBsAg, hepatitis B surface antigen; HBcrAg, hepatitis B core-related antigen; anti-HBc IgG, immunoglobulin G antibody to hepatitis B core antigen; COI, cut-off index.

**Table 4:** Structural features of the HDV amber/W site at baseline.

	PTZ_ID	HDV Genotype	A-C Mismatch	n. of DRBM	Length
Viral responder	HDV02_TV	1e	YES	3	4-8-6
	HDV04_OI	1e	YES	3	4-8-6
	HDV06_GE	1b	NO	3	4-8-7
	HDV07_BL	1e	NO	3	4-8-7
	HDV08_ZYJ	1b	YES	3	4-8-8
	HDV09_IN	1e	YES	4	4-8-3-6
	HDV012_BG	1e	YES	3	4-8-8
	HDV013_IDM	1e	YES	3	4-8-8
	HDV016_PG	1e	YES	3	4-8-6
	OHDV02_CL	1e	YES	3	4-8-8
	OHDV06_PG	UND *	YES	4	4-8-4-3
	OHDV07_RG	1e	YES	3	4-8-7
	OHDV08_SY	1e	YES	3	4-8-6
	HDV014_PN	1e	YES	3	4-8-6
Viral non-responder	HDV01_SR	1e	YES	3	4-8-6
	HDV03_GA	1e	YES	3	4-8-6
	HDV05_GC	1e	YES	3	4-8-6
	HDV010_KI	1e	NO	3	4-8-6
	HDV015_GS	1e	YES	3	4-8-8
	OHDV01 _AMD	1e	YES	3	4-8-7
	OHDV03_GG	1c	YES	4	4-4-3-8
	OHDV04_MP	1e	YES	3	4-7-6
	OHDV05_MAA	1e	YES	3	4-8-6
VB	HDV011_KO	1e	YES	3	4-8-6

* UND, GT1 subtype undetermined. Amber/W site of HDV antigenome RNA secondary structure in 24 HBV/HDV coinfected patients who achieved 48 weeks of treatment. Specifically, 14 were viral responders and 10 were viral non-responders (including 1 patient with virological breakthrough). VB, viral breakthrough.

## Data Availability

Sequences were submitted to GenBank (accession numbers: PQ514005 and PQ673658-PQ673680). The data presented in this study are available on request from the corresponding author.

## Supplementary Materials

The following supporting information can be downloaded at: https://www.mdpi.com/article/10.3390/biomedicines13020280/s1, Figure S1: HDV RNA kinetics for (A) 16 virological responders, (B) 12 virological non-responders, and (C) 2 patients with virological breakthrough while receiving bulevirtide treatment at different time points; Table S1: Characteristics of biochemical and virological variables at baseline and during bulevirtide treatment in virological responders; Table S2: Characteristics of biochemical and virological variables at baseline and during bulevirtide treatment in virological non-responders; Figure S2: Unrooted phylogenetic tree with non-collapsed lineages; Table S3: Summary of baseline polymorphisms detected in the functional domains of delta antigen (HDAg) protein among v-responders and v-non-responders; Figure S3: Comparison of amino acid polymorphisms in the functional domains of delta antigen (HDAg) protein among virological responders (v-responders) and virological non-responders (v-non-responders) patients; Figure S4: Predicted HDV antigenome RNA secondary structures from the full-length baseline genome sequences for 24 patients receiving bulevirtide treatment. Secondary structure predictions were performed using the RNAfold WebServer (http://rna.tbi.univie.ac.at//cgi-bin/RNAWebSuite/RNAfold.cgi, accessed on 23 September 2024). The colored bar represents base-pair probabilities.

## Abbreviations

ADAR1	Adenosine Deaminase Acting on RNA 1
ALT	Alanine Aminotransferase
AST	Aspartate Aminotransferase
BL	Baseline
BLV	Bulevirtide
BMI	Body Mass Index
CCS	Coiled-coil sequence
cccDNA	covalently closed circular DNA
CLEIA	Chemiluminescence Enzyme Immunoassay
COI	Cut-off index
cp	copies
DRBM	Double-stranded RNA binding motifs
HBsAg	Hepatitis B Virus surface antigen
HBcAb	Hepatitis B core antibody
HBcrAg	Hepatitis B core-related antigen
HBeAg	Hepatitis B e antigen
HBV	Hepatitis B virus
HCC	Hepatocellular carcinoma
HDV	Hepatitis Delta virus
HDV RNA	Hepatitis Delta virus RNA
HIV	Human Immunodeficiency Virus
HCV	Hepatitis C Virus
HDAg	Hepatitis Delta Antigen
IQR	Interquartile range
IU/mL	International Units per milliliter
LLOD	Lower Limit of Detection
L-HDAg	Large Hepatitis Delta Antigen
MCMC	Markov Chain Monte Carlo
MFE	Minimum free energy
NES	Nuclear Export Signal
NLS	Nuclear Localization Signal
NTCP	Sodium taurocholate cotransporting polypeptide
NUC	Nucleos(t)ide-analogues
PCR	Polymerase Chain Reaction
RBD	RNA Binding Domain
RNP	Ribonucleoprotein
S-HDAg	Small Hepatitis Delta Antigen
SVR	Sustained virological response
TW	Time Week
VAS	Virus-assembly signal
