# Supplementary material for: Exploring Predictive Factors for Bulevirtide Treatment Response in Hepatitis Delta-Positive Patients
Source: Biomedicines. 2025 Jan 23;13(2):280. doi: 10.3390/biomedicines13020280 (PMC11852621; doi:10.3390/biomedicines13020280)
Supplement: Supplementary file 1 [file biomedicines-13-00280-s001.zip › FigureS4_HDV secondary structure.pdf]

Figure S4: Predicted HDV antigenome RNA secondary structures from the full-length baseline genome sequences for 24 patients receiving bulevirtide treatment. Secondary structure predictions were performed using the RNAfold WebServer (http://rna.tbi.univie.ac.at//cgi-bin/RNAWebSuite/RNAfold.cgi, accessed on September 23, 2024). The colored bar represents base-pair probabilities.

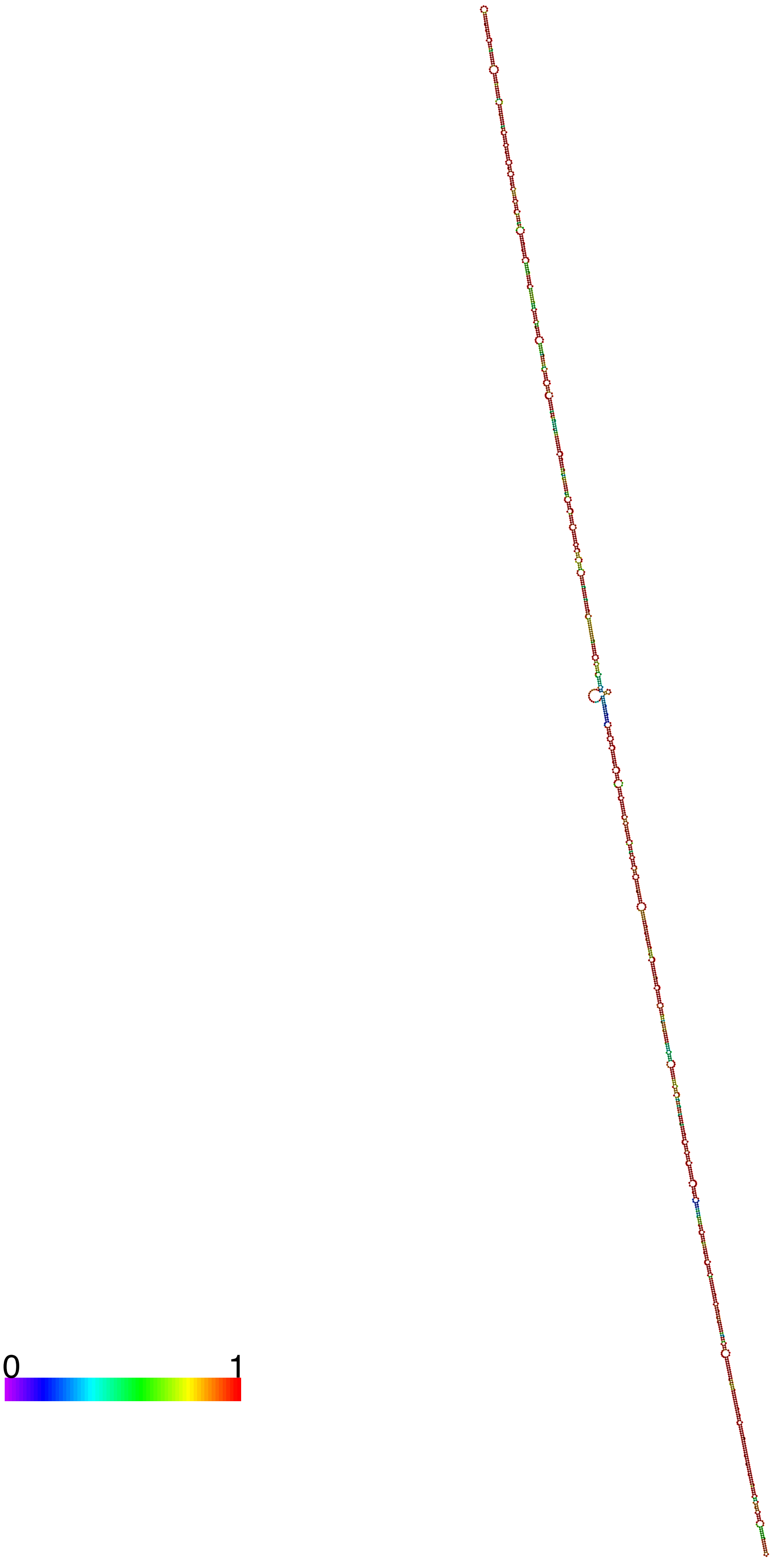

0
1

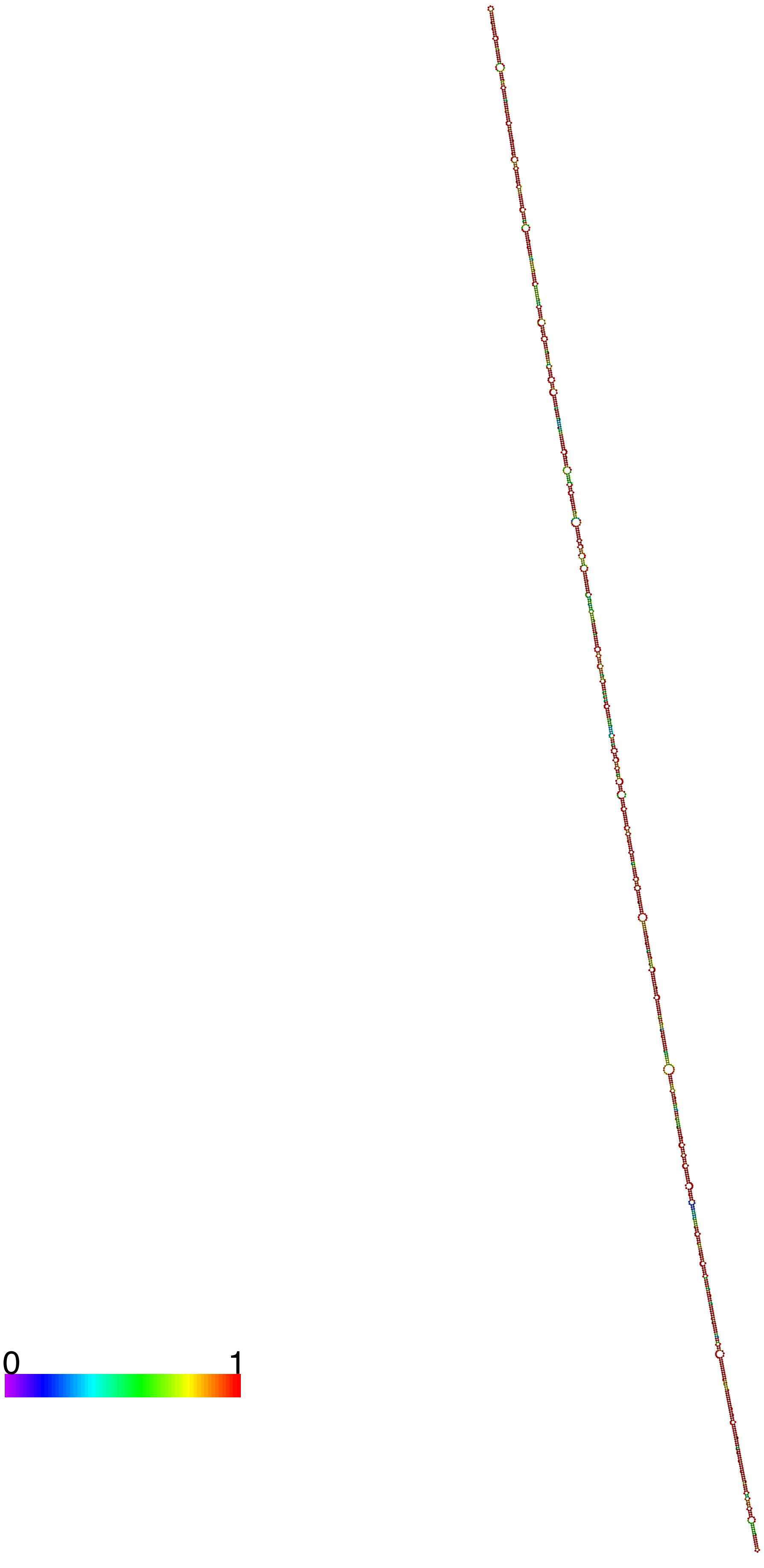
0
1

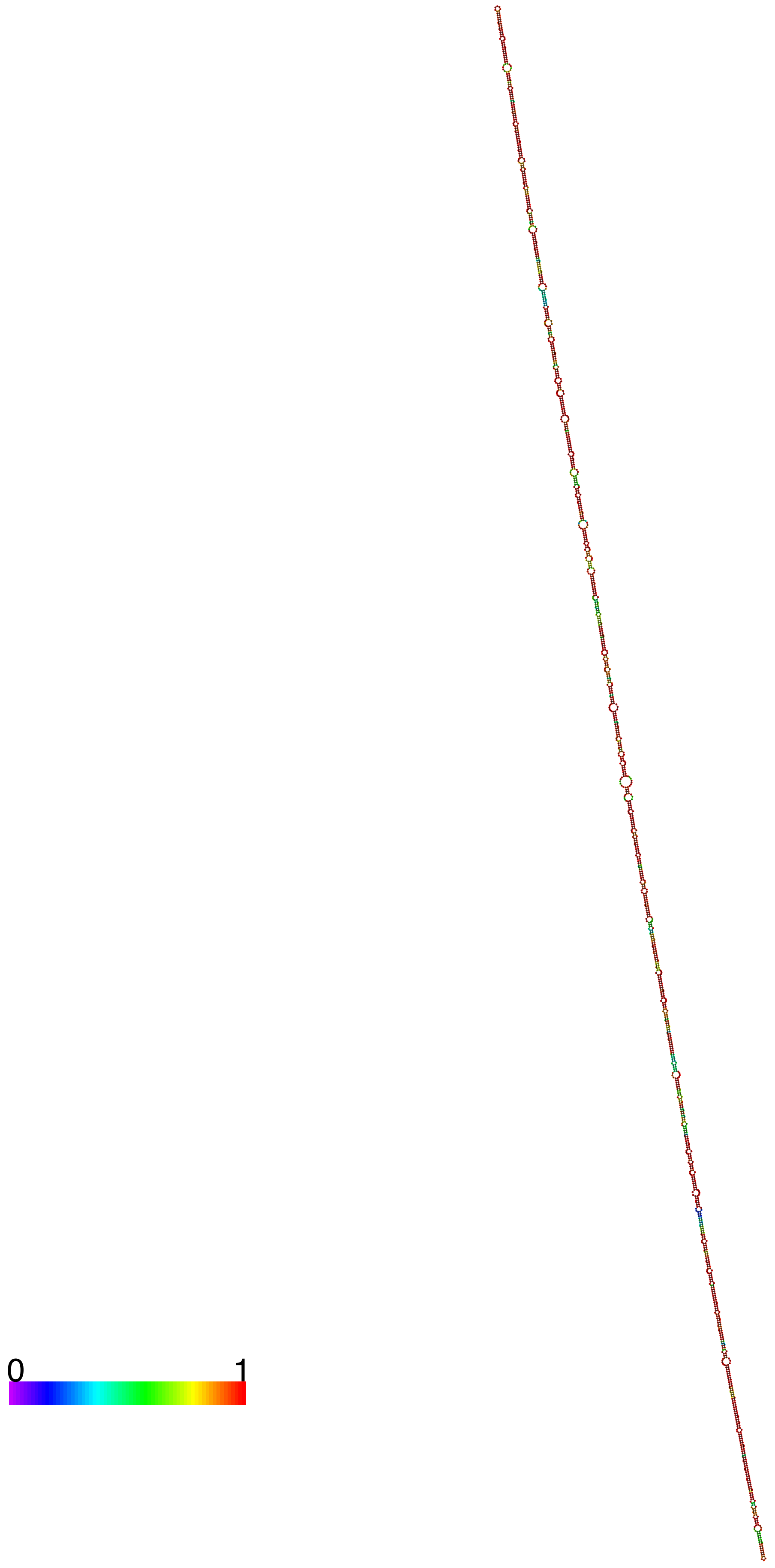
0
1

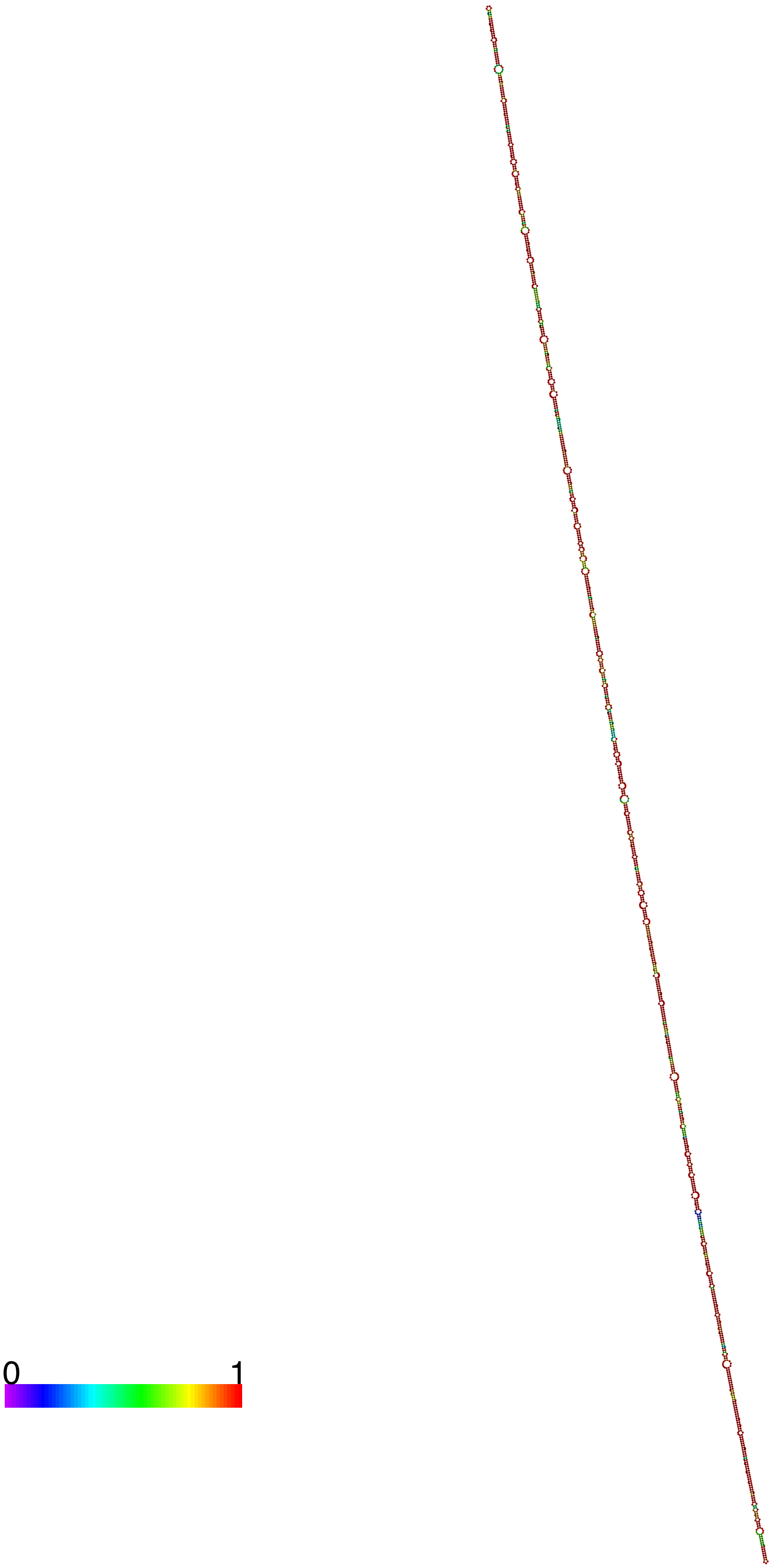

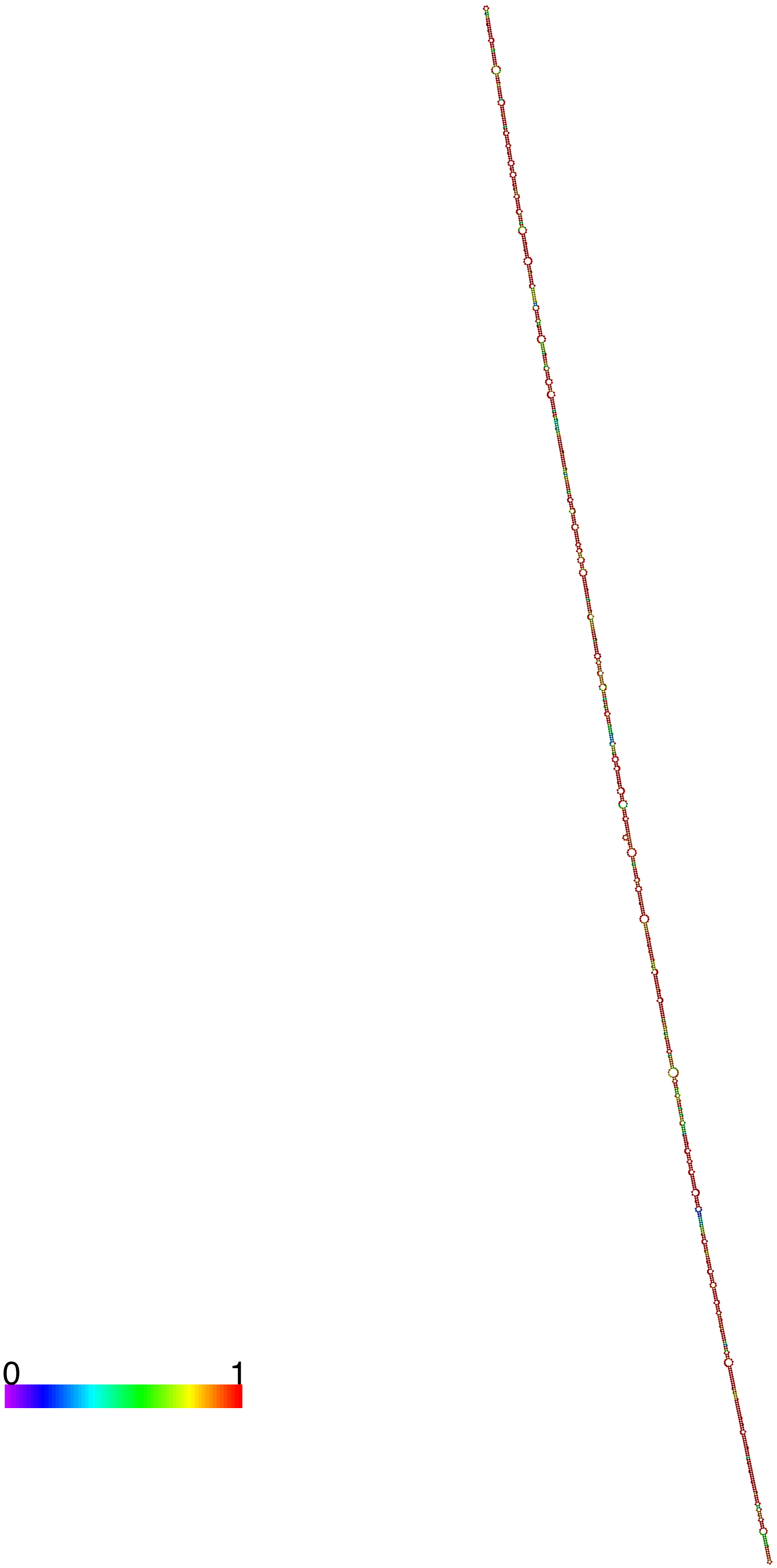
0
1

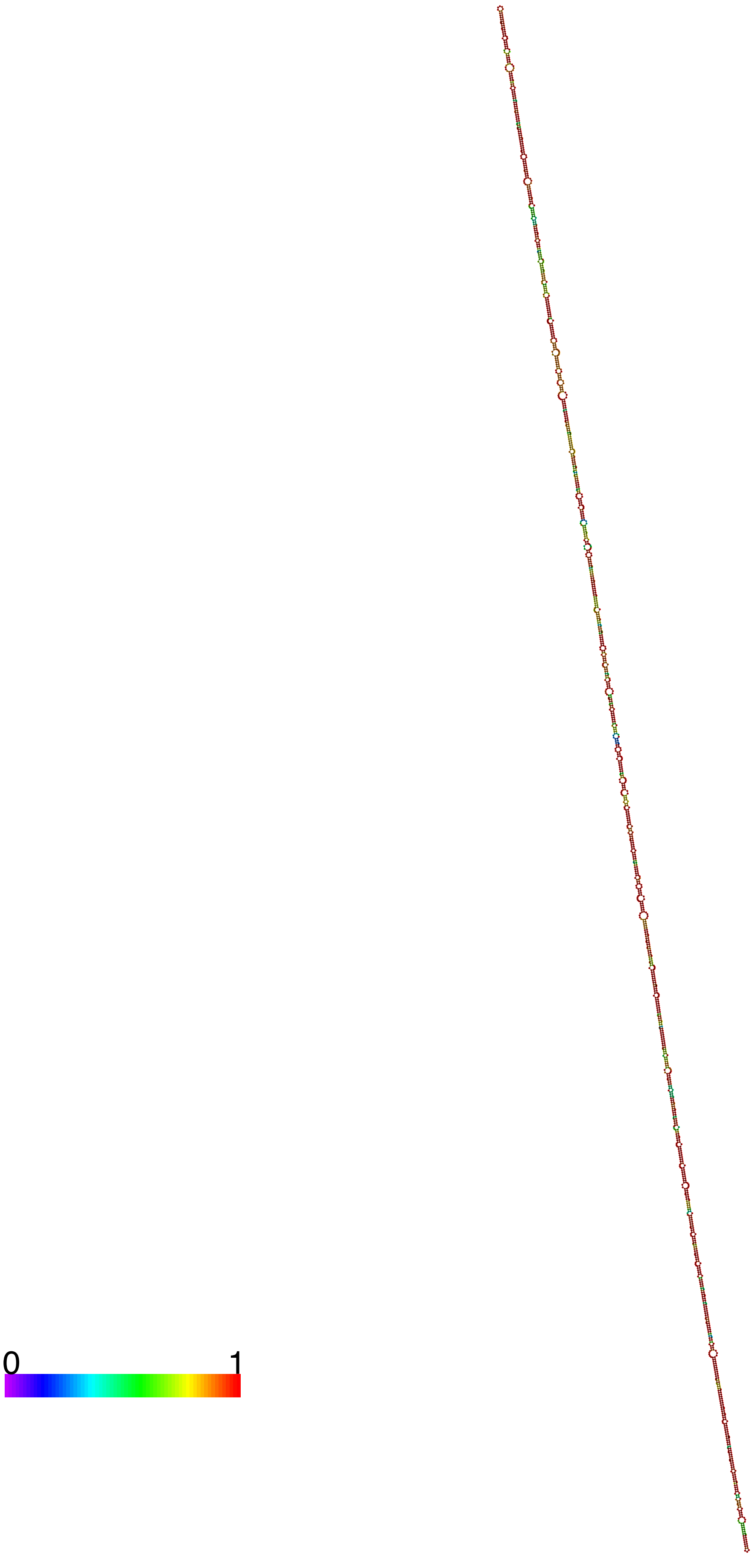
0
1

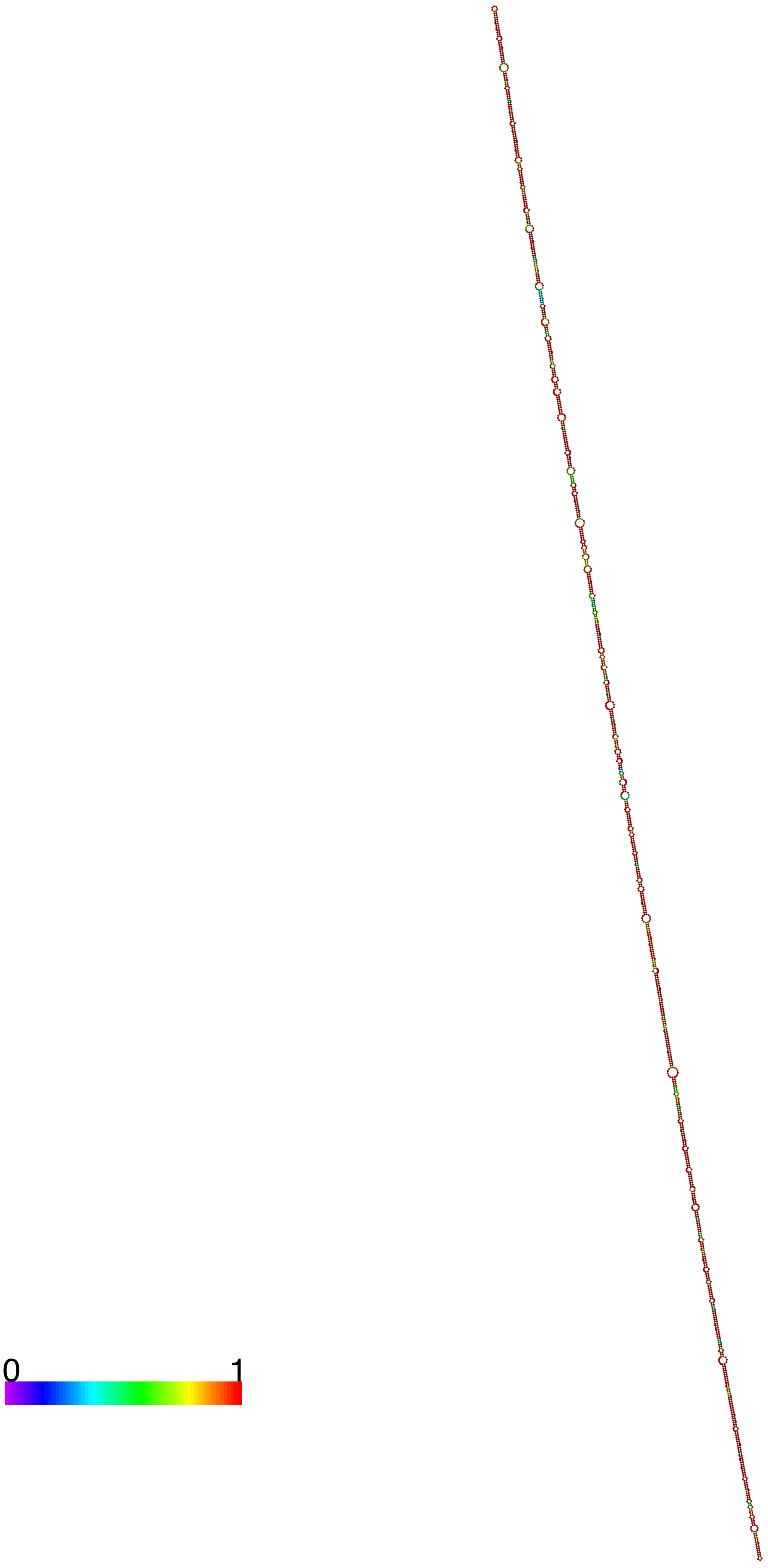

0
1

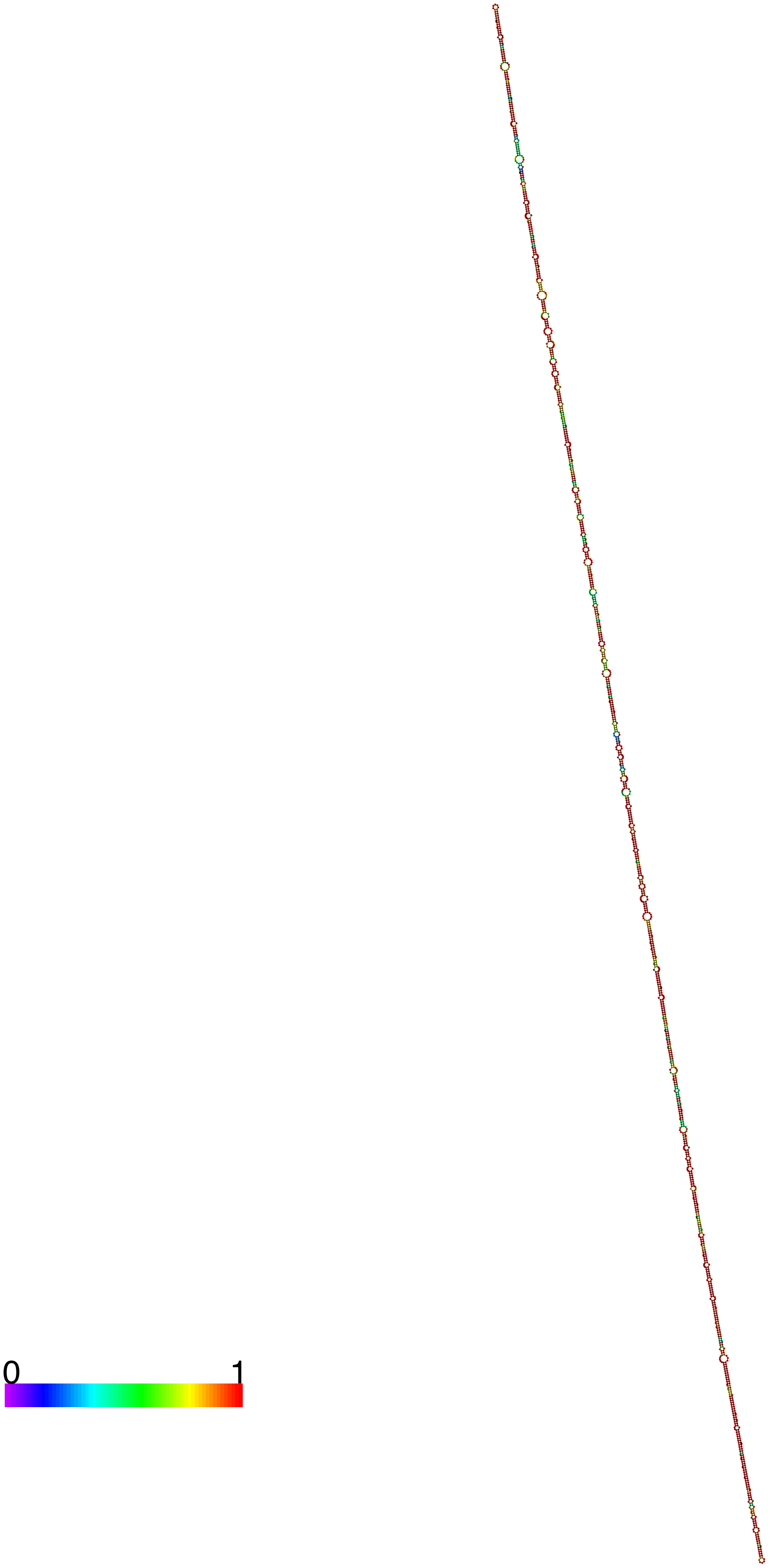
0
1

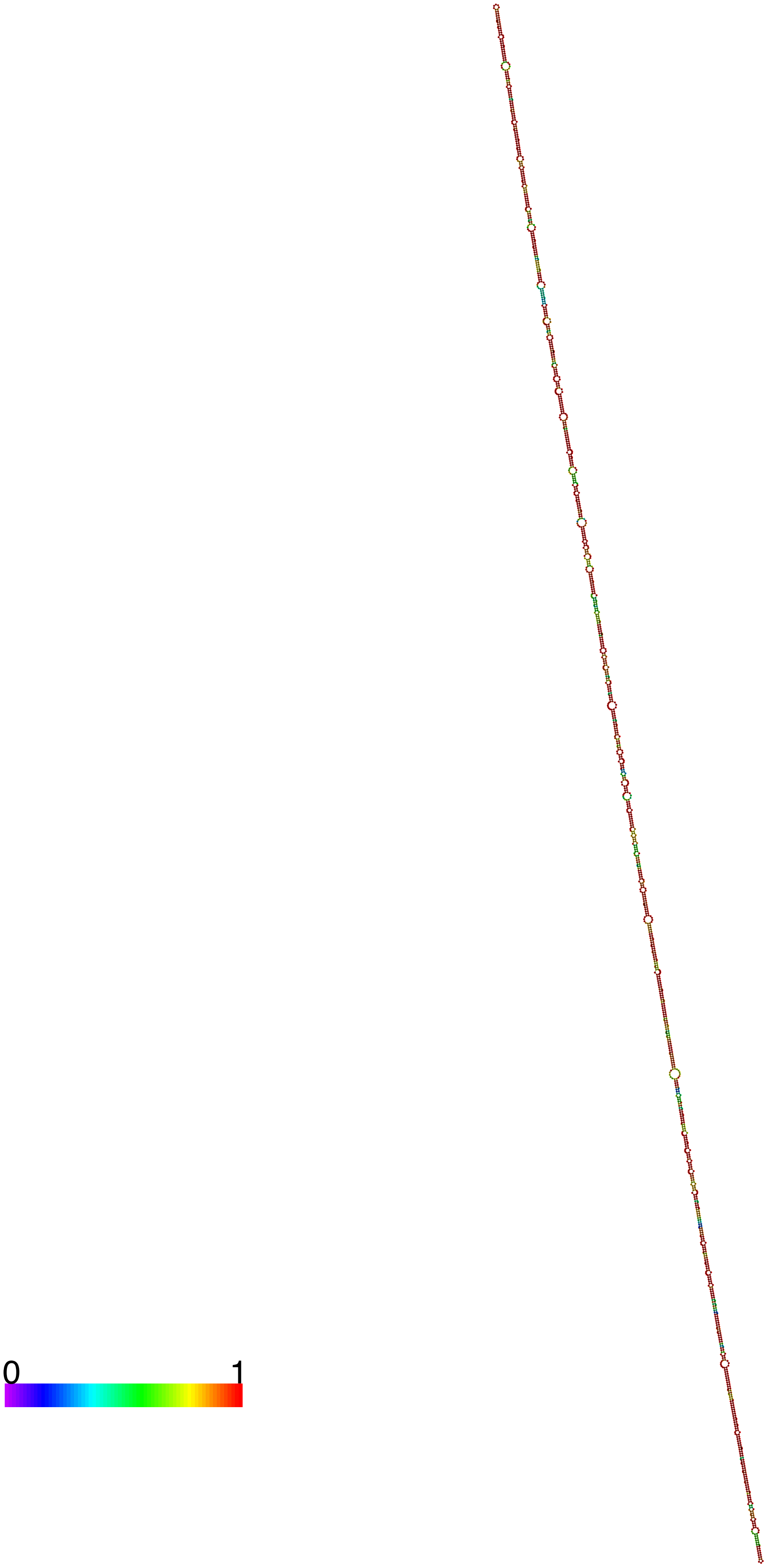
0
1

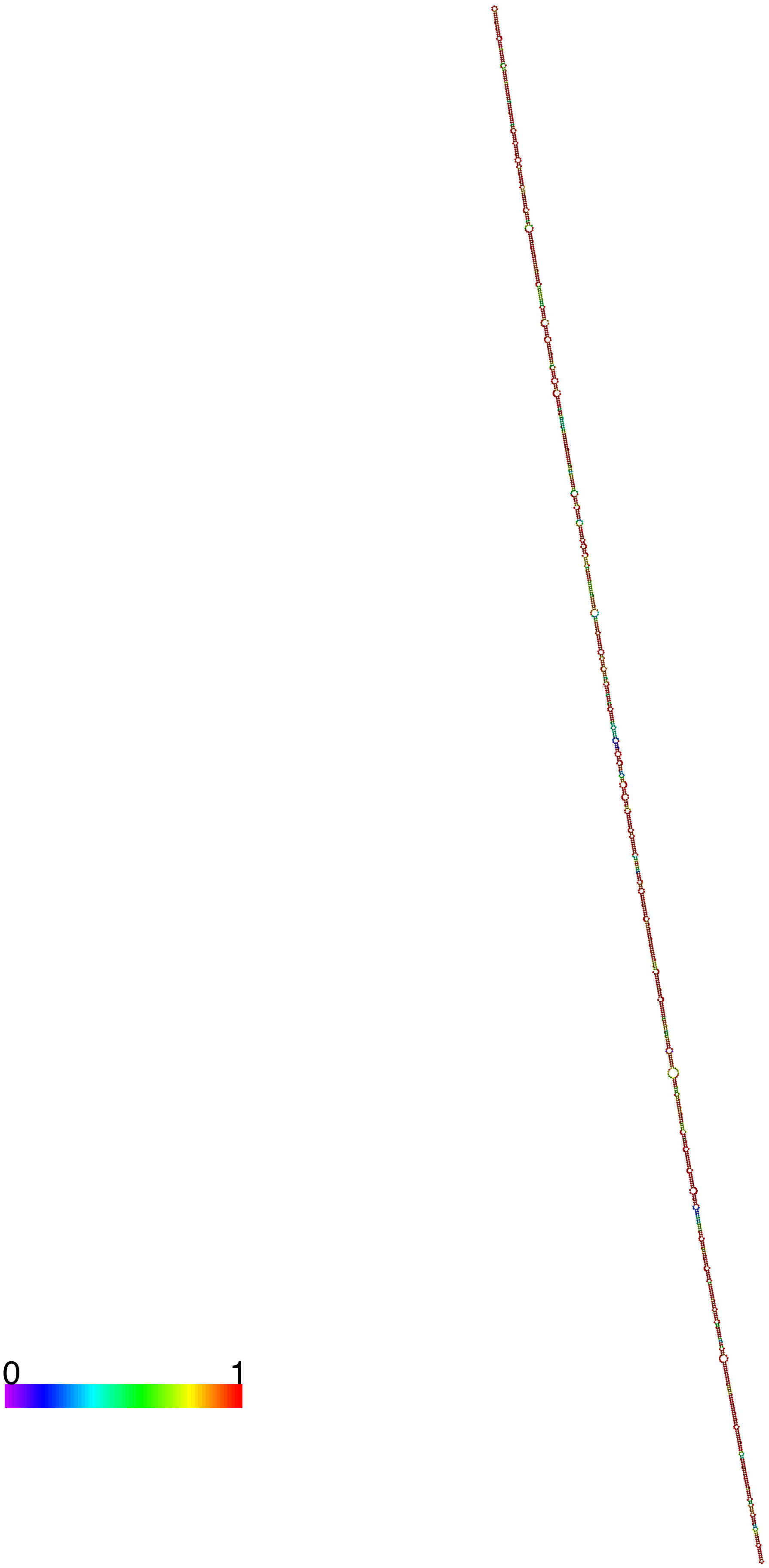

0
1

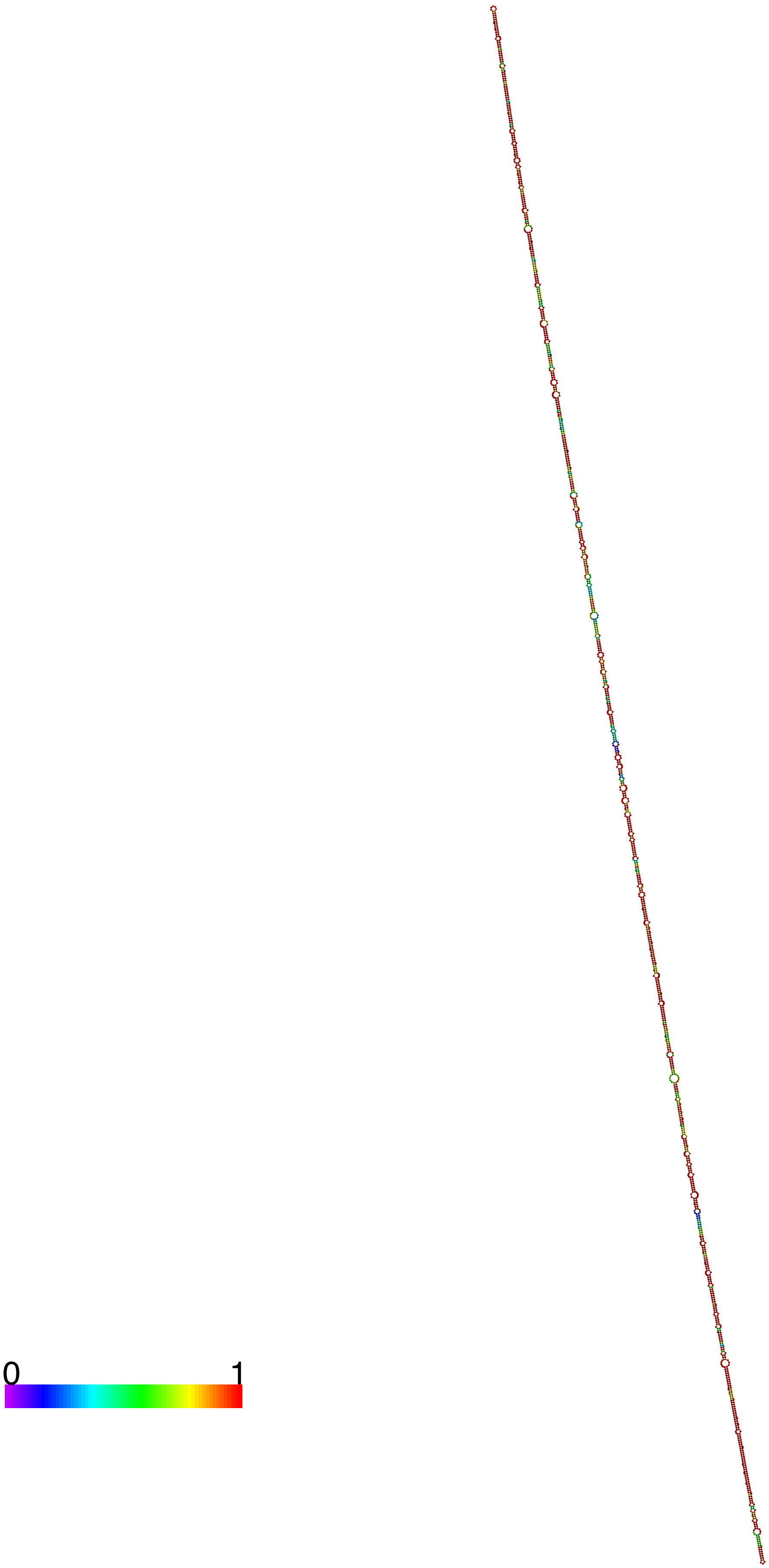
0
1

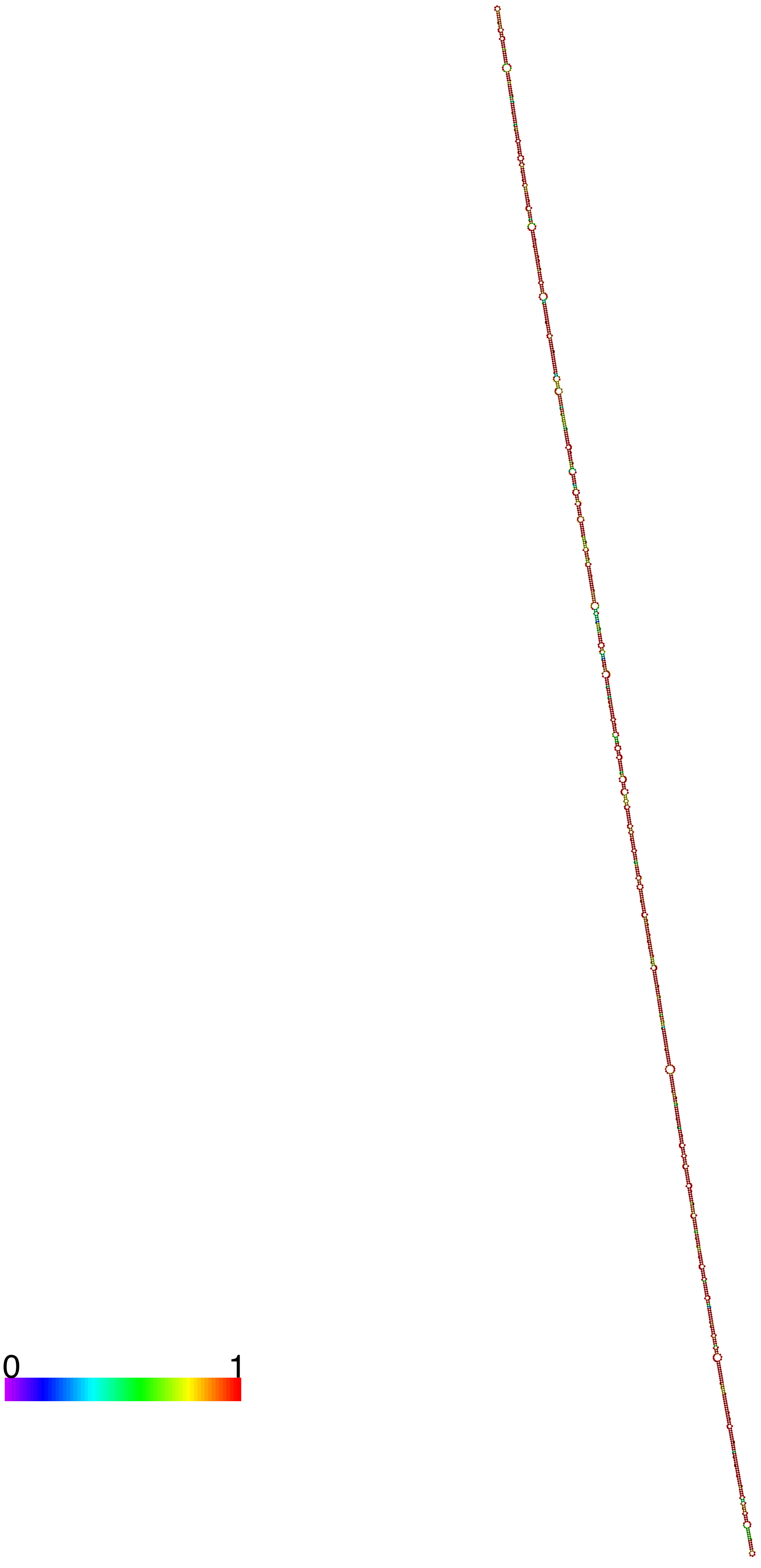
0
1

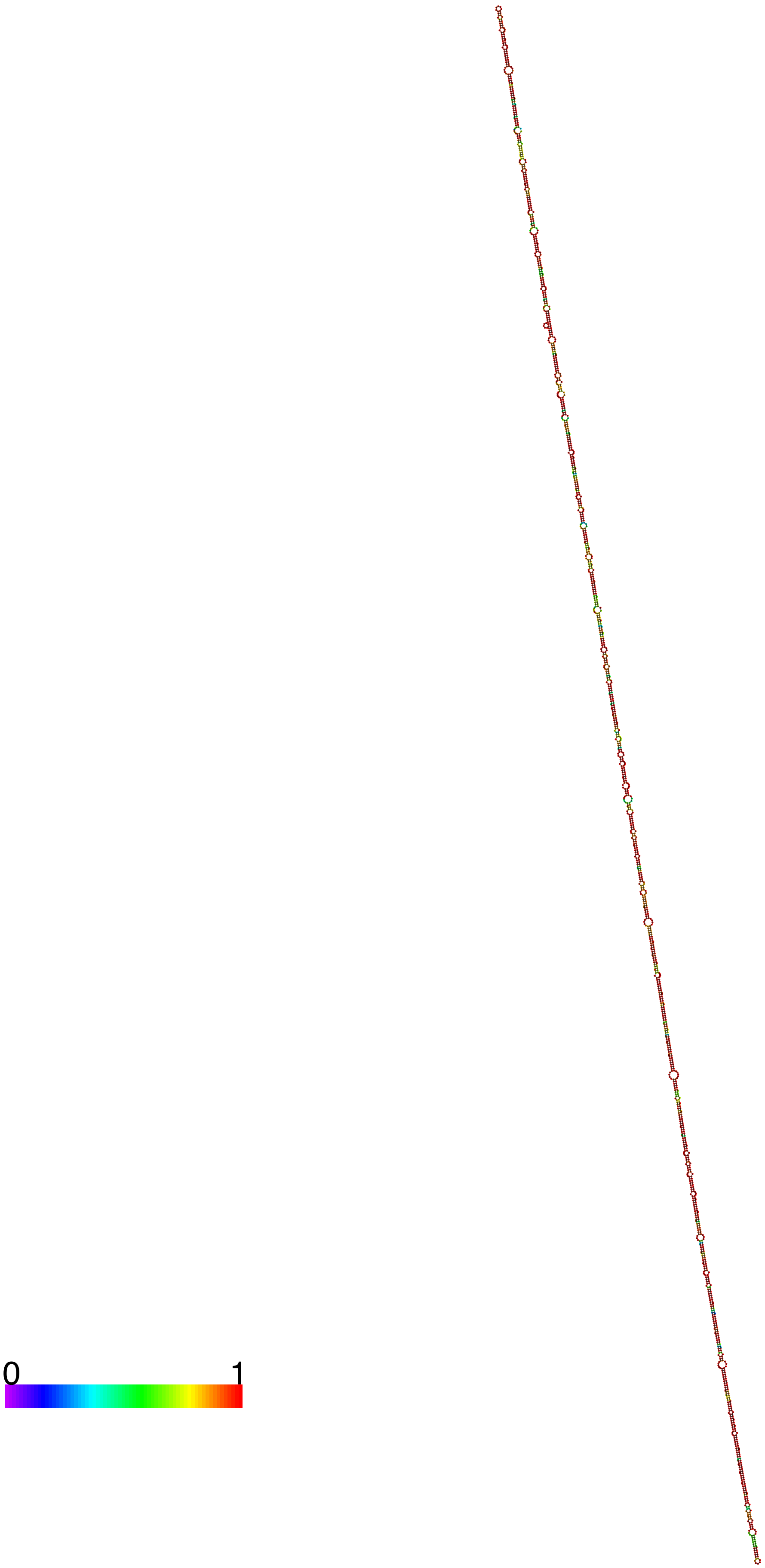

0
1

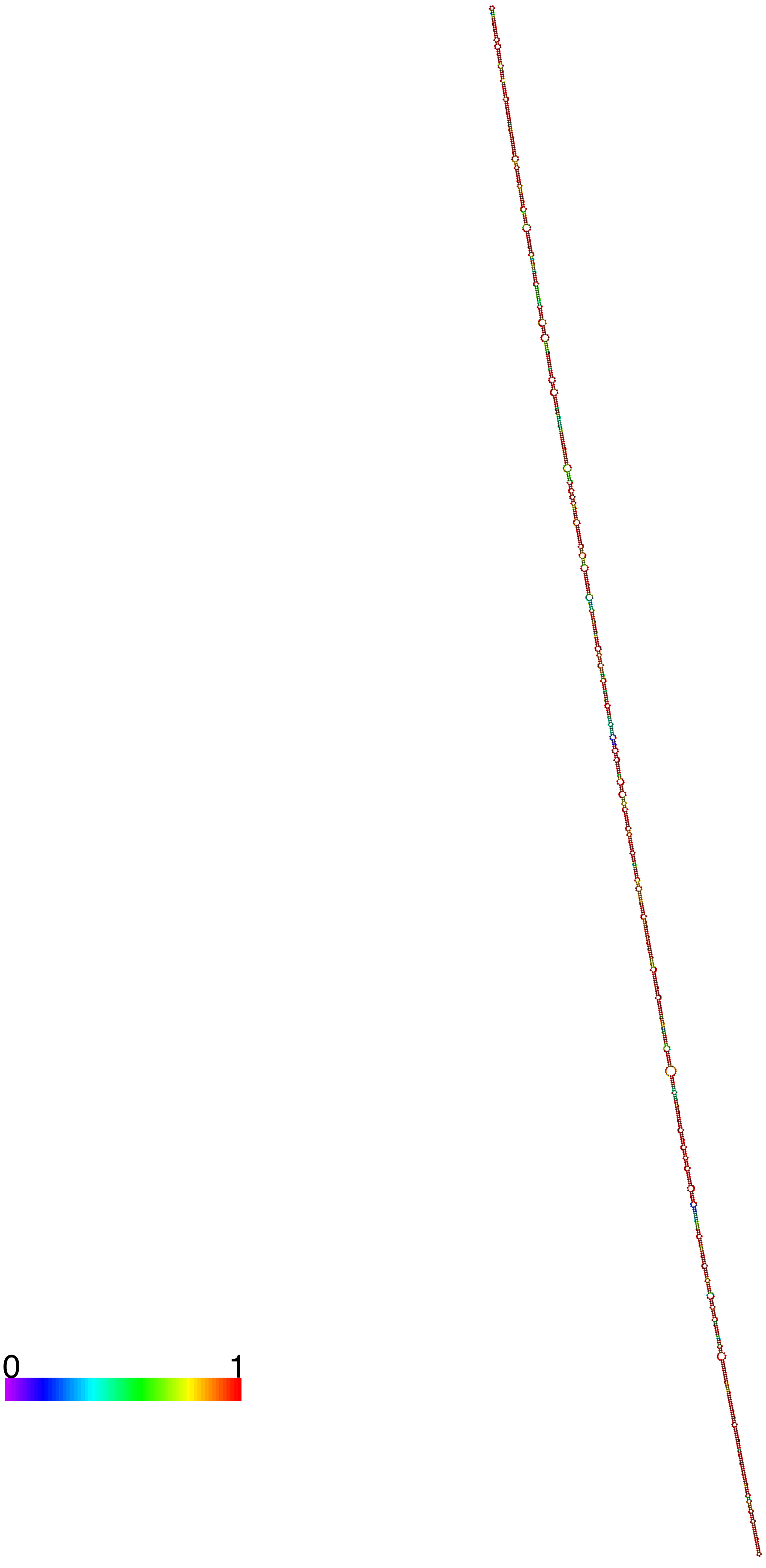
0
1

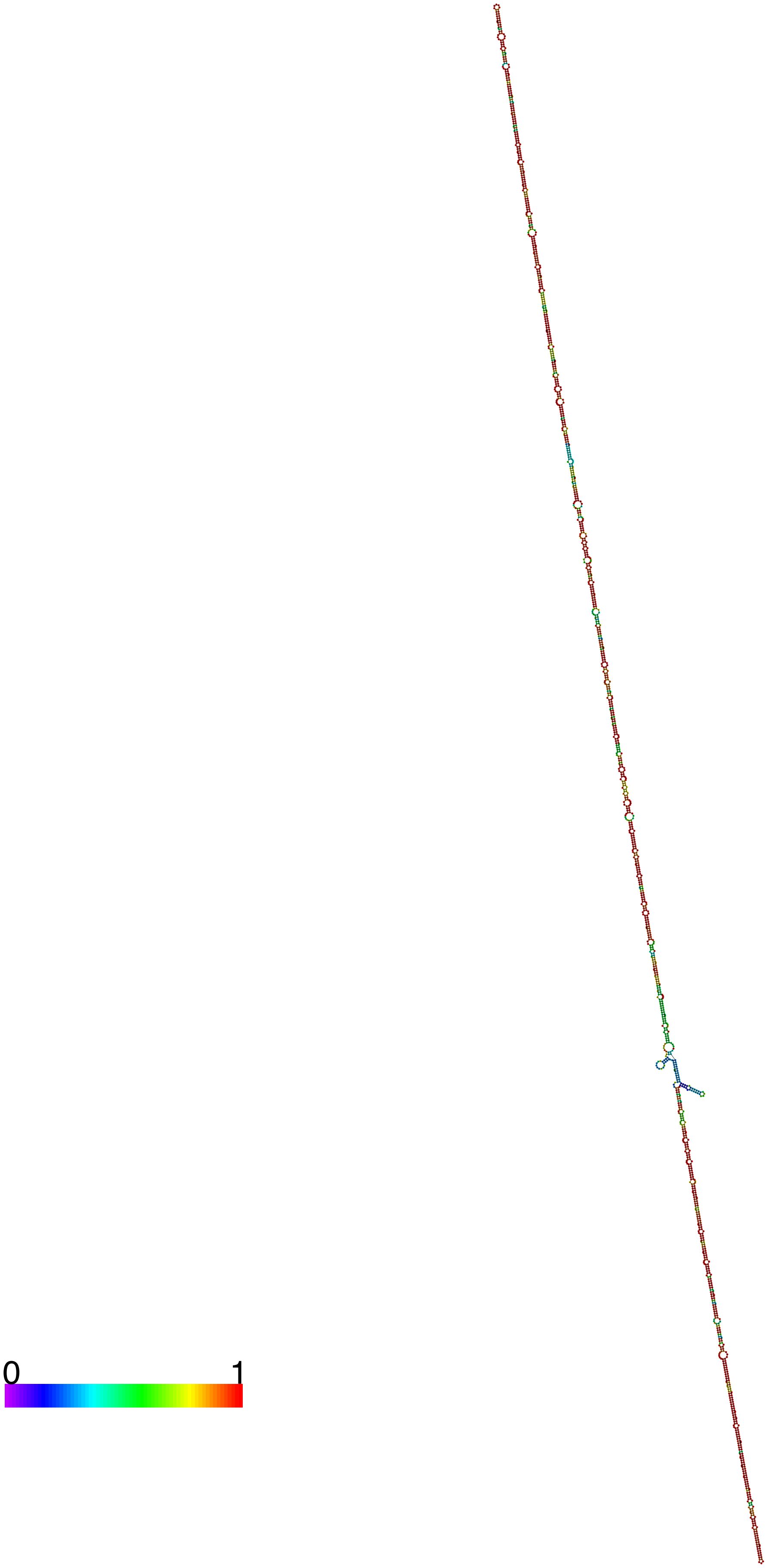
0
1

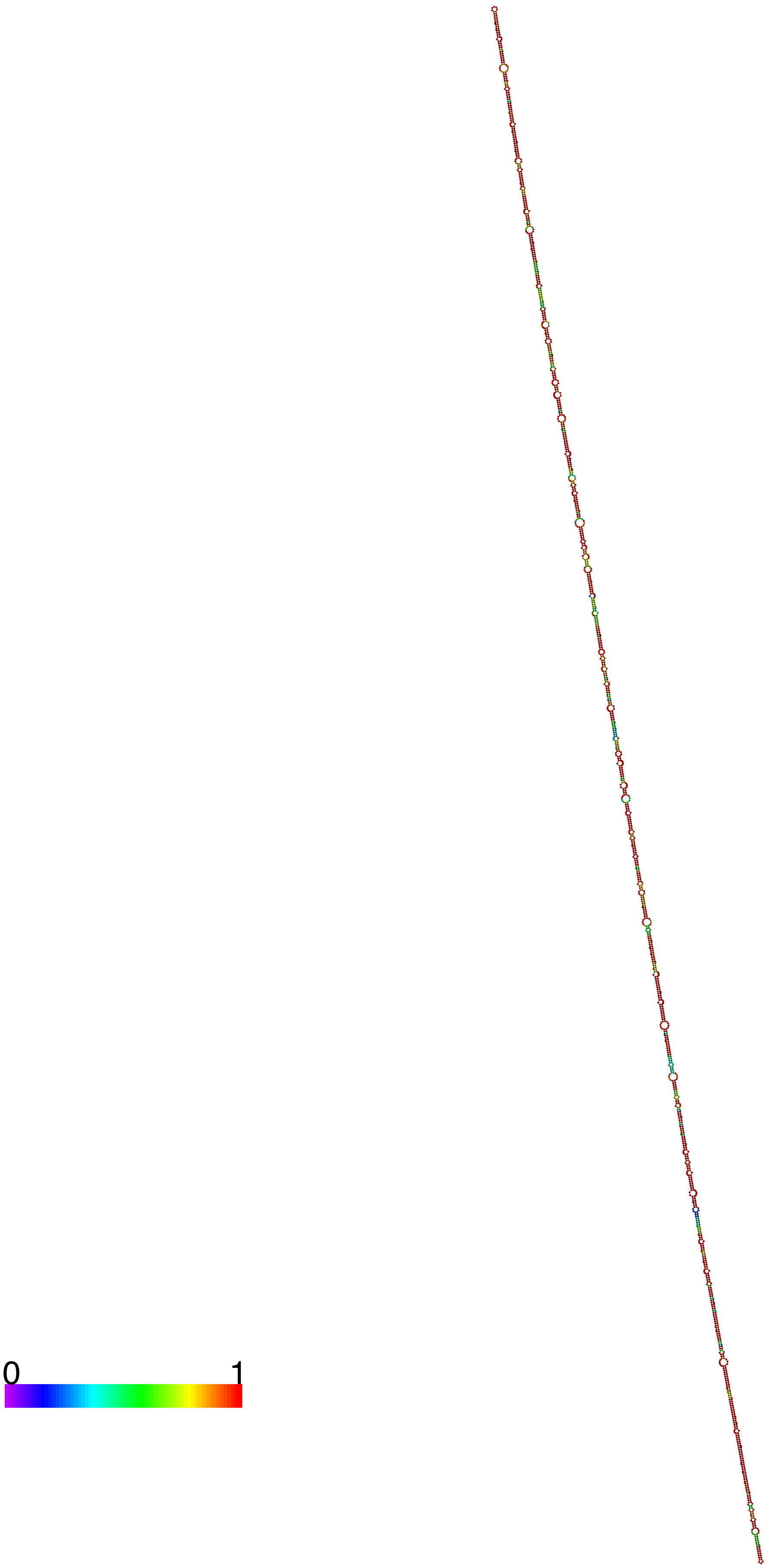
0
1

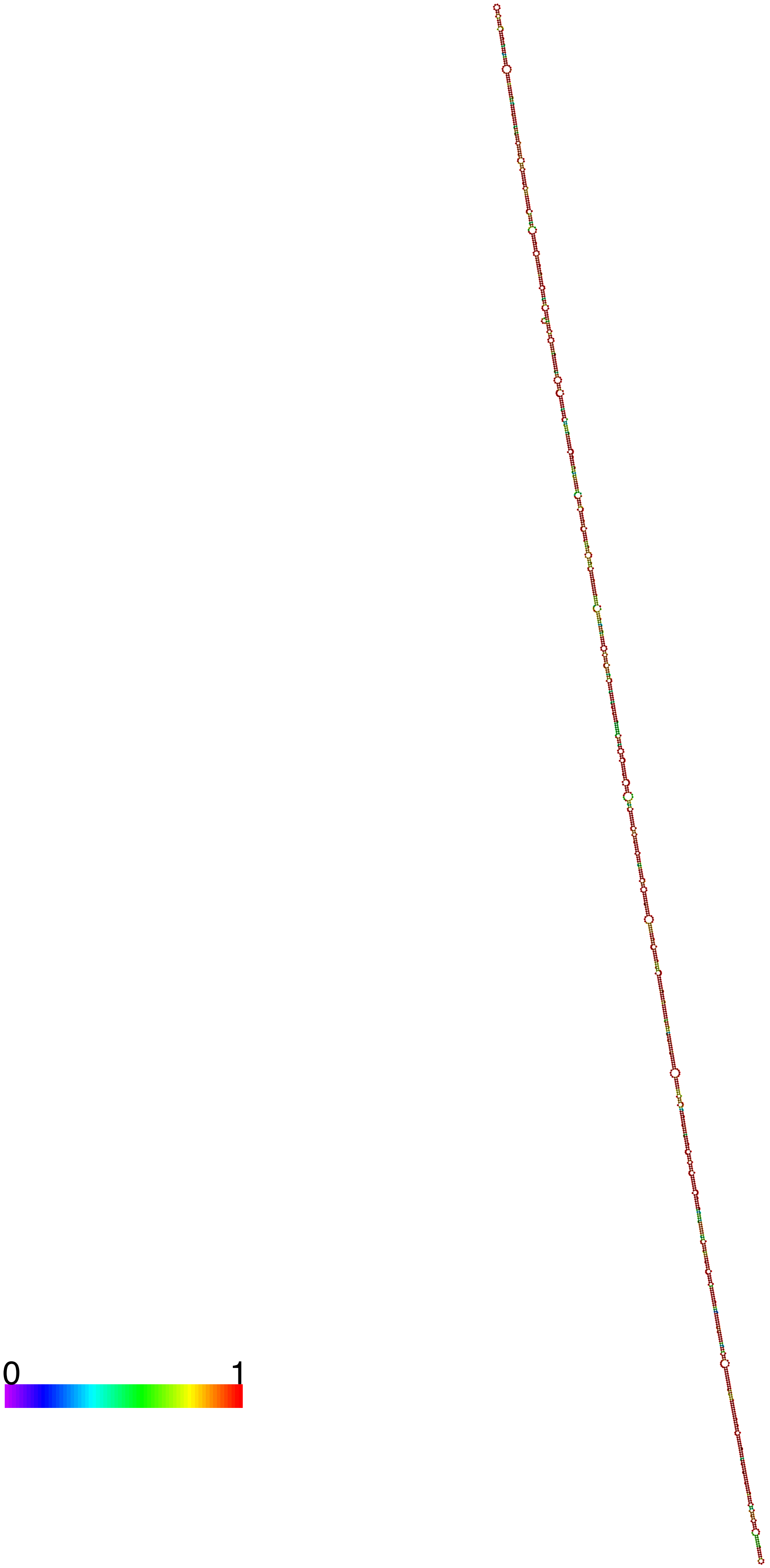
0
1

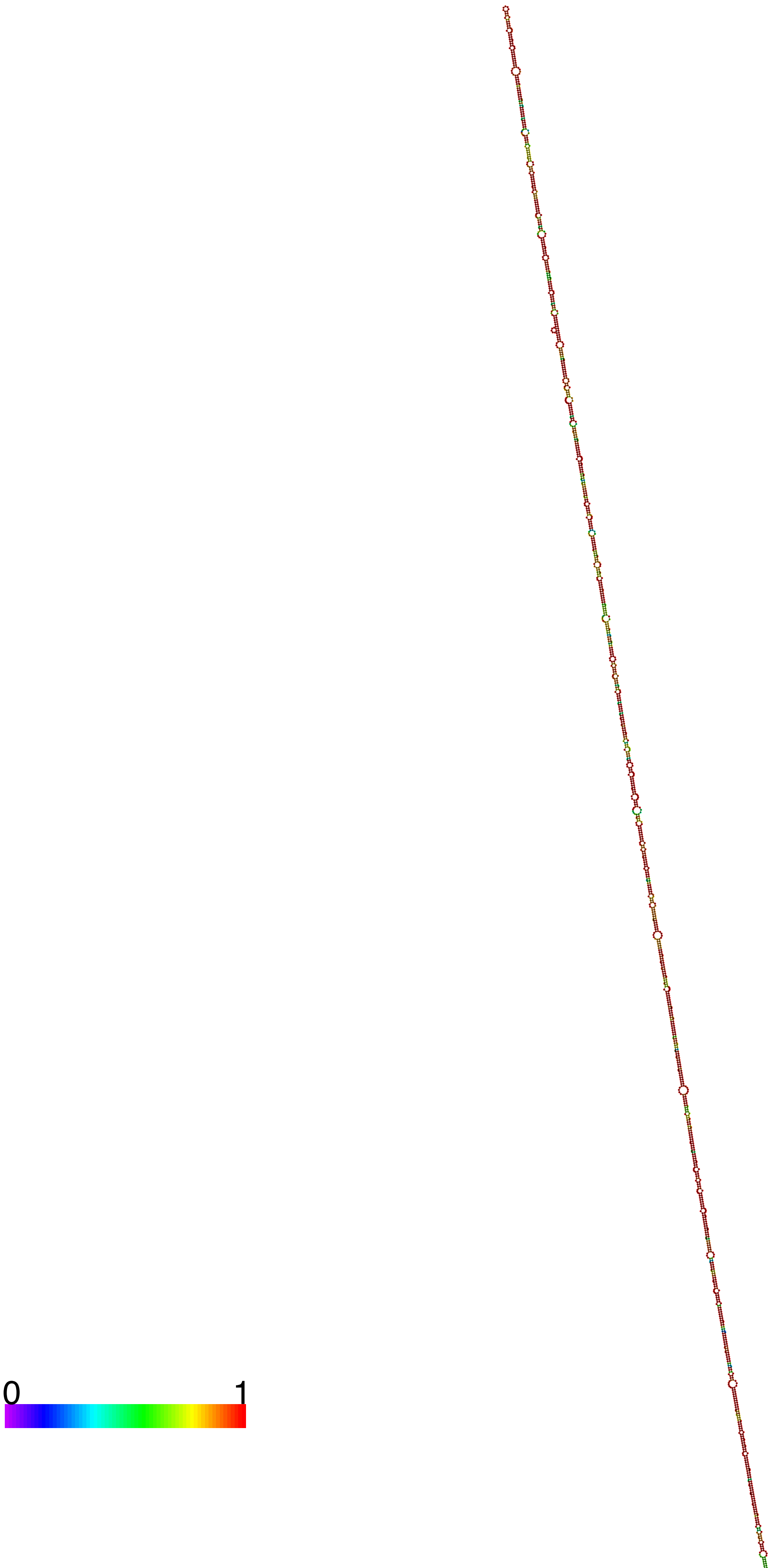
0
1

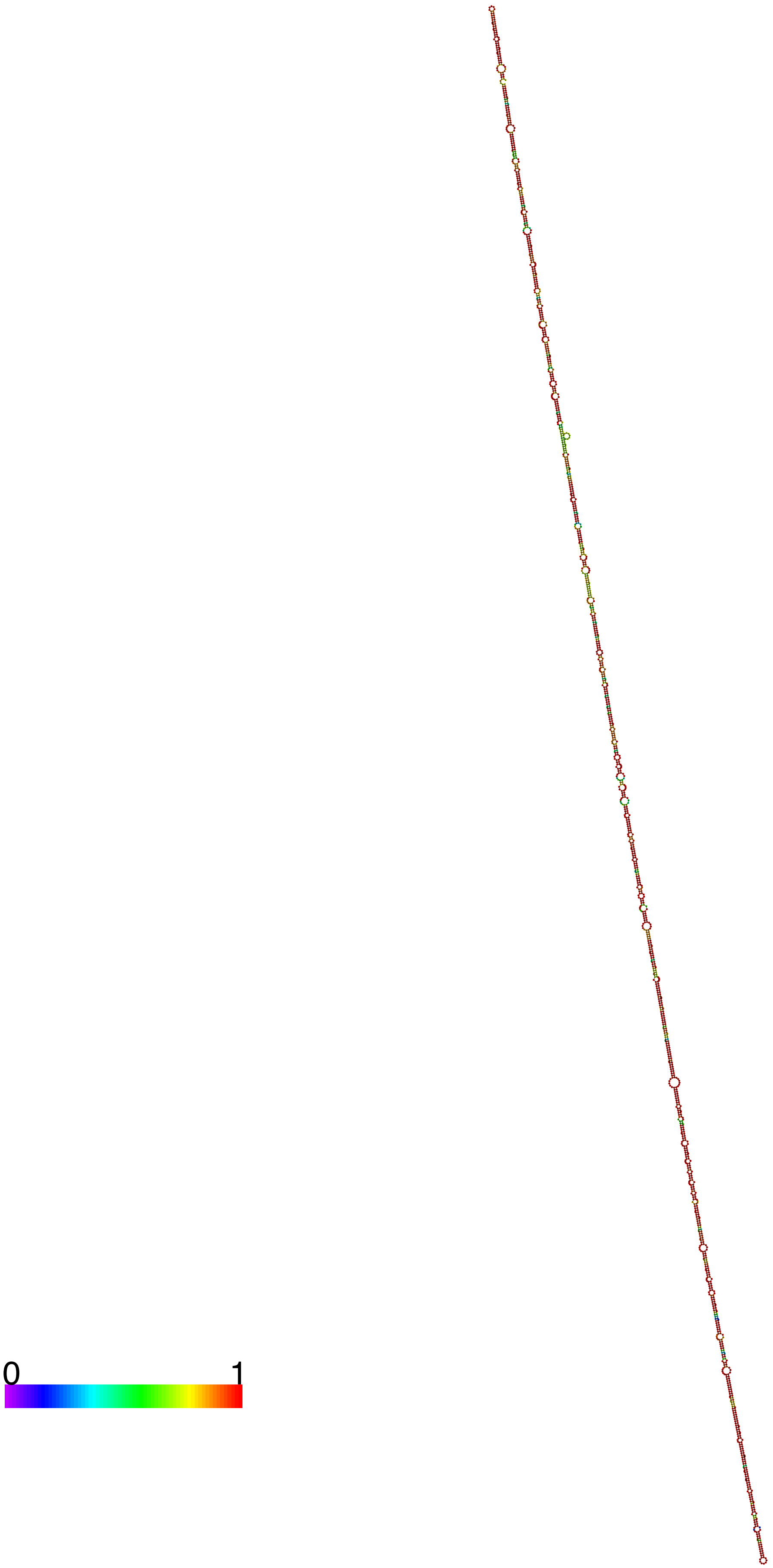
0
1

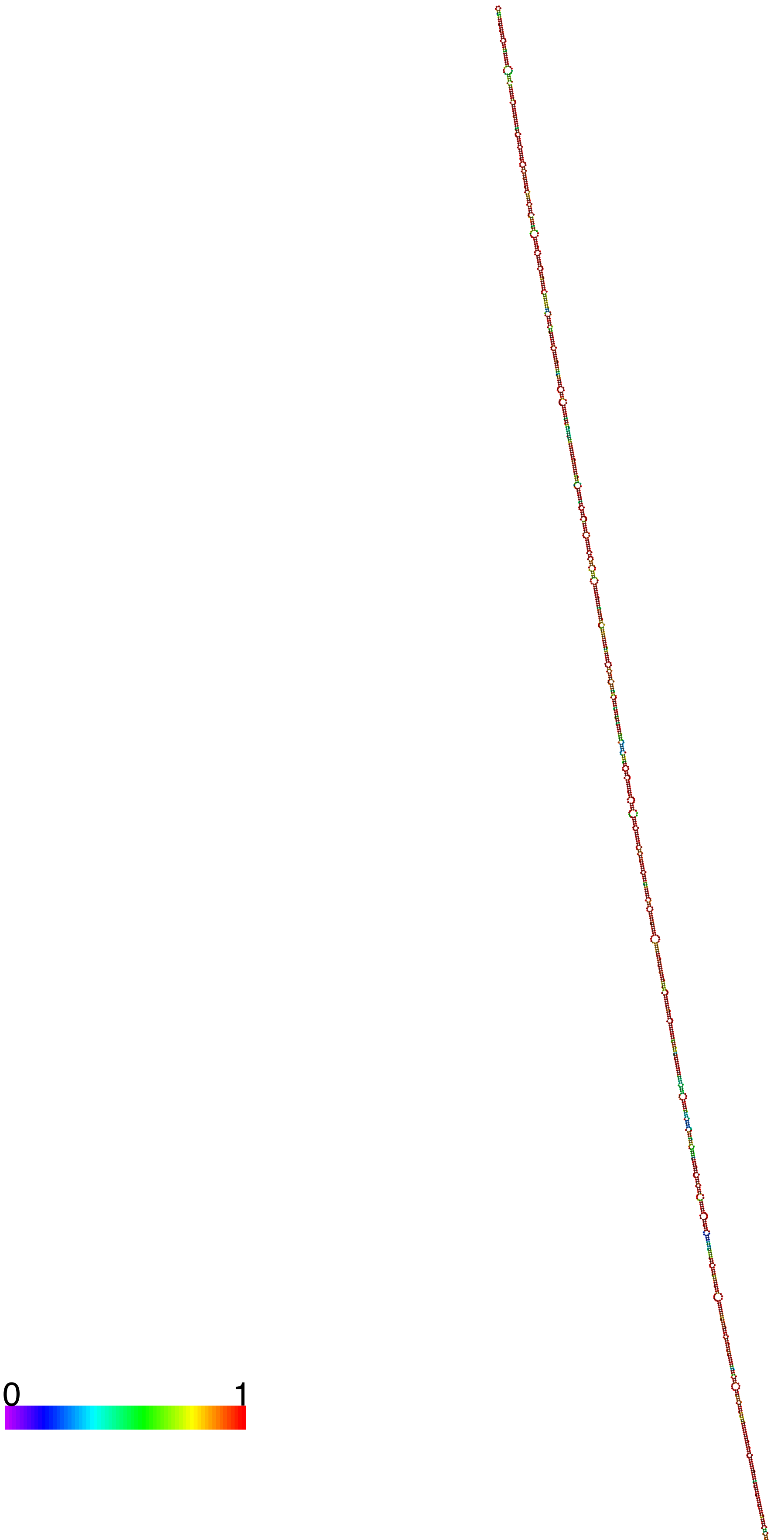
0
1

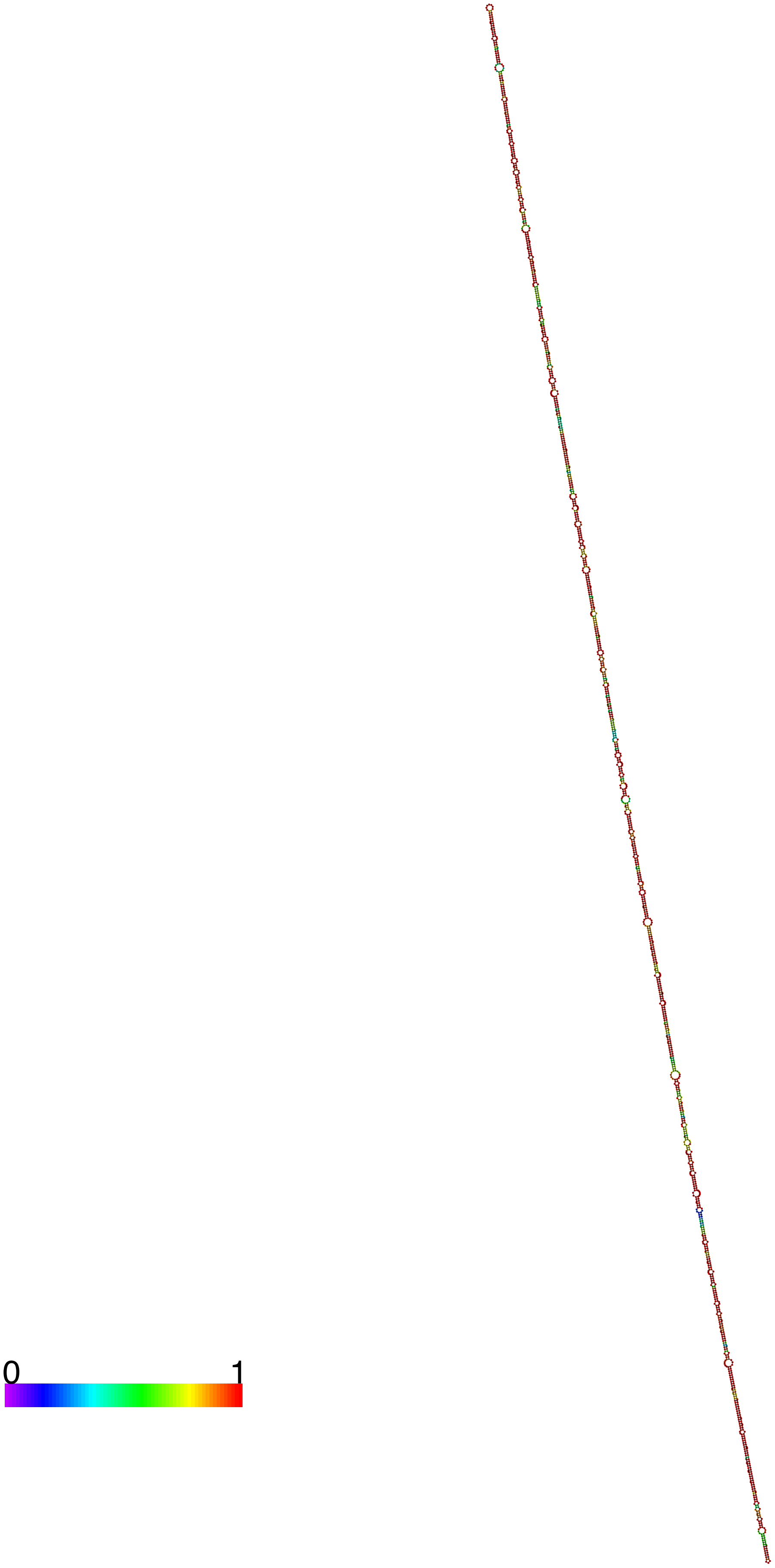
0
1

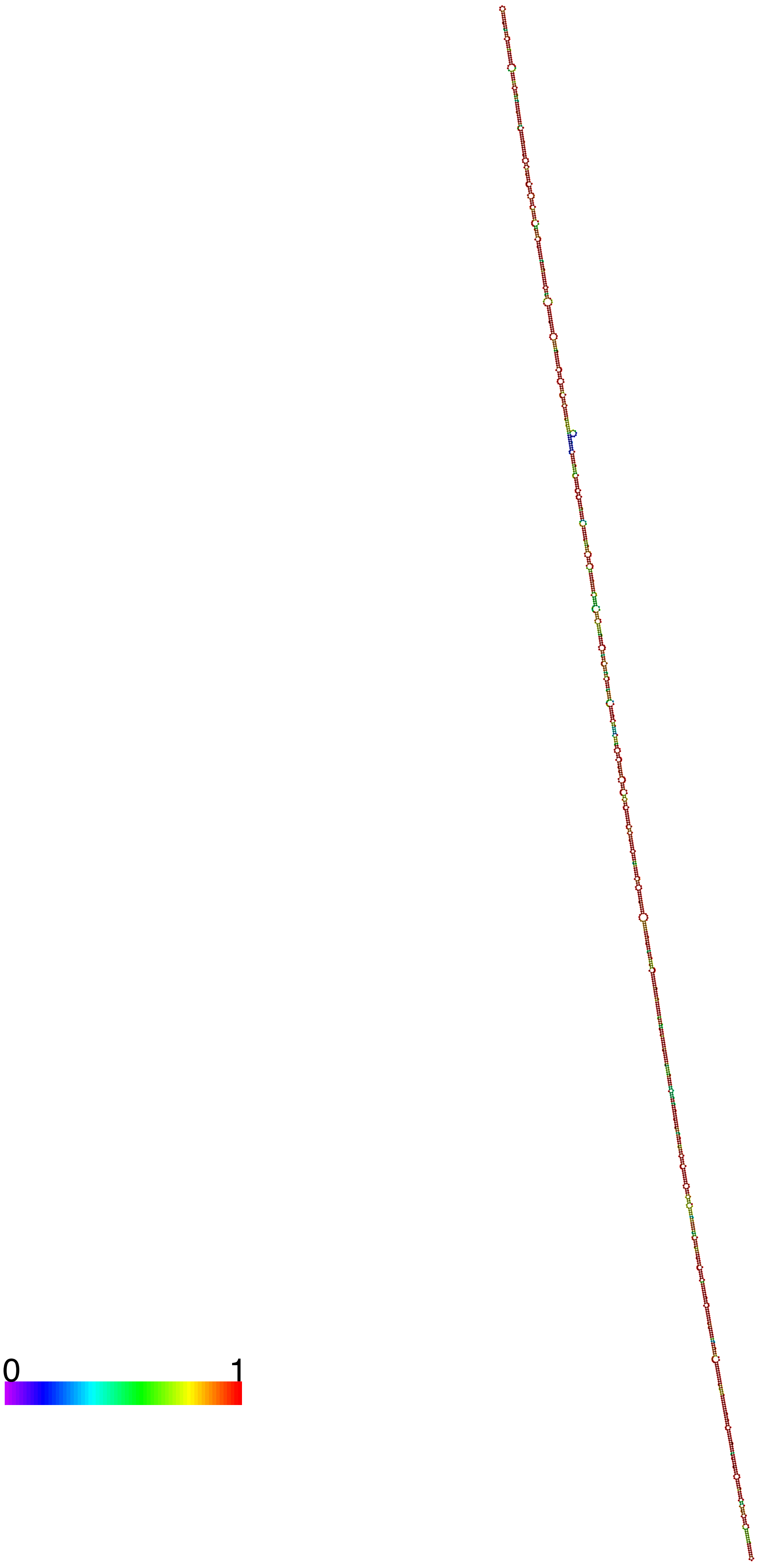

0
1

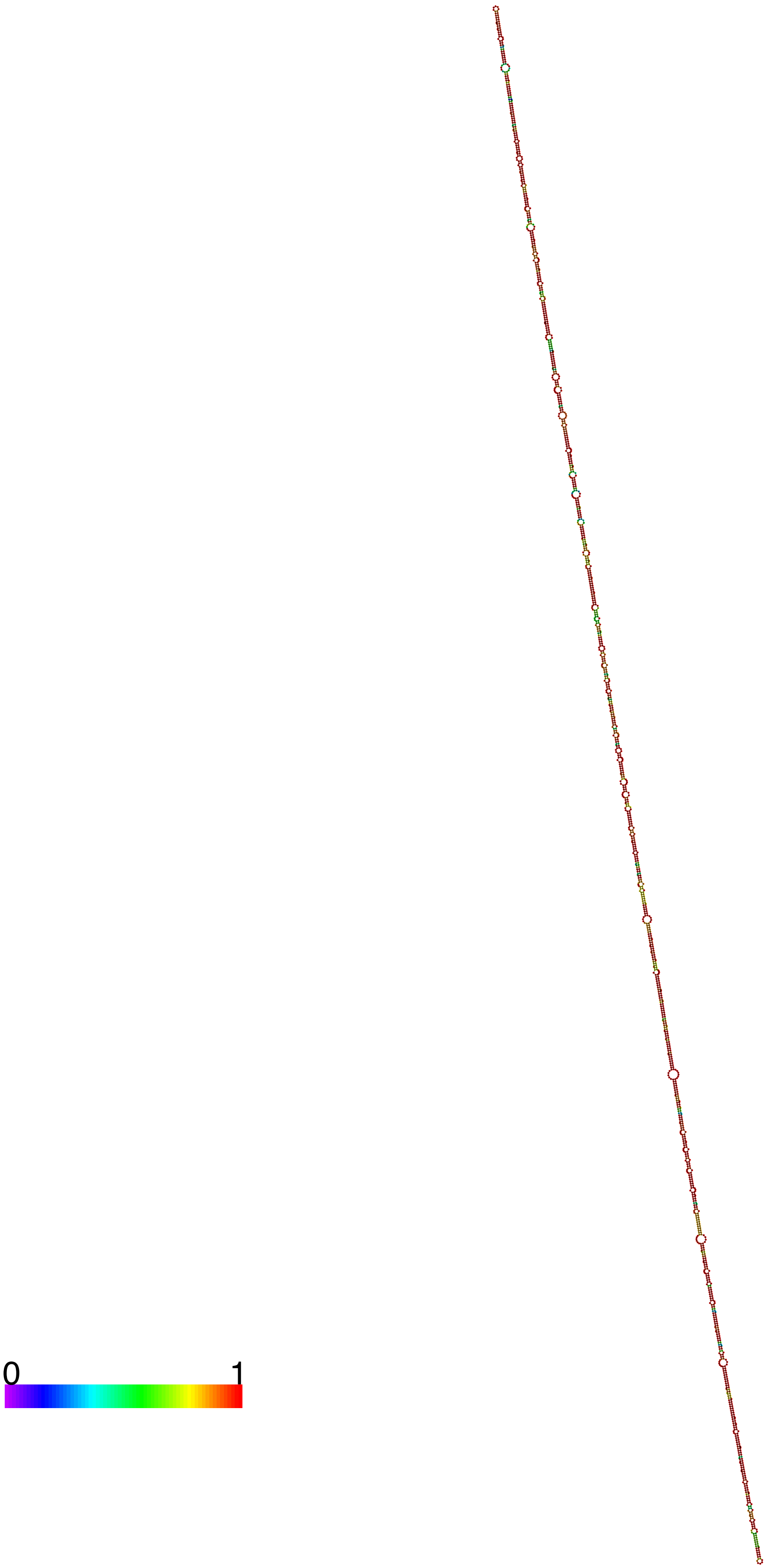
0
1

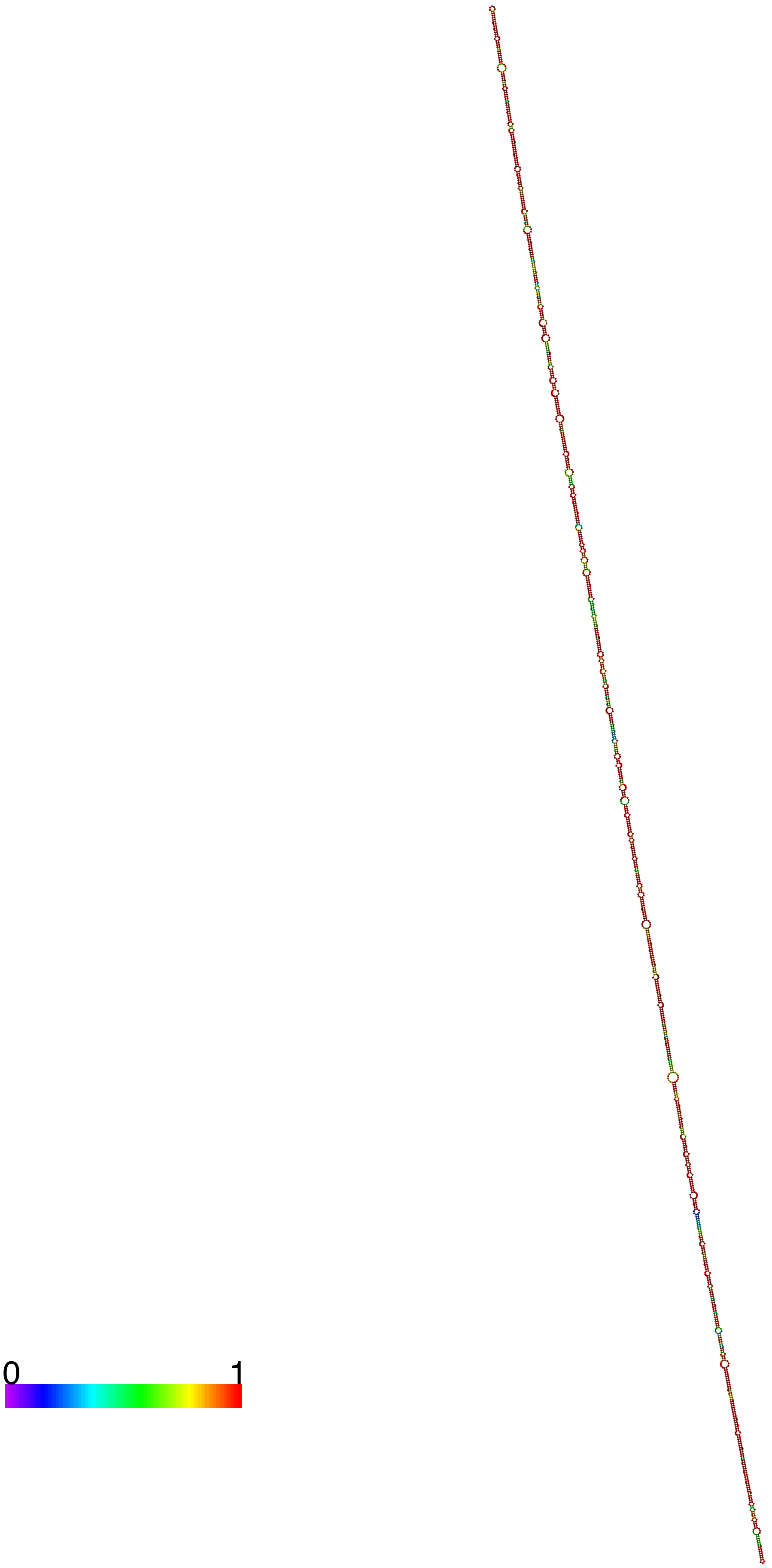

0
1
