# Supplementary material for: Exploring Predictive Factors for Bulevirtide Treatment Response in Hepatitis Delta-Positive Patients
Source: Biomedicines. 2025 Jan 23;13(2):280. doi: 10.3390/biomedicines13020280 (PMC11852621; doi:10.3390/biomedicines13020280)
Supplement: Supplementary file 1 [file biomedicines-13-00280-s001.zip › REV1_HDV_BLV_Supplementary materials.pdf]

Supplementary materials

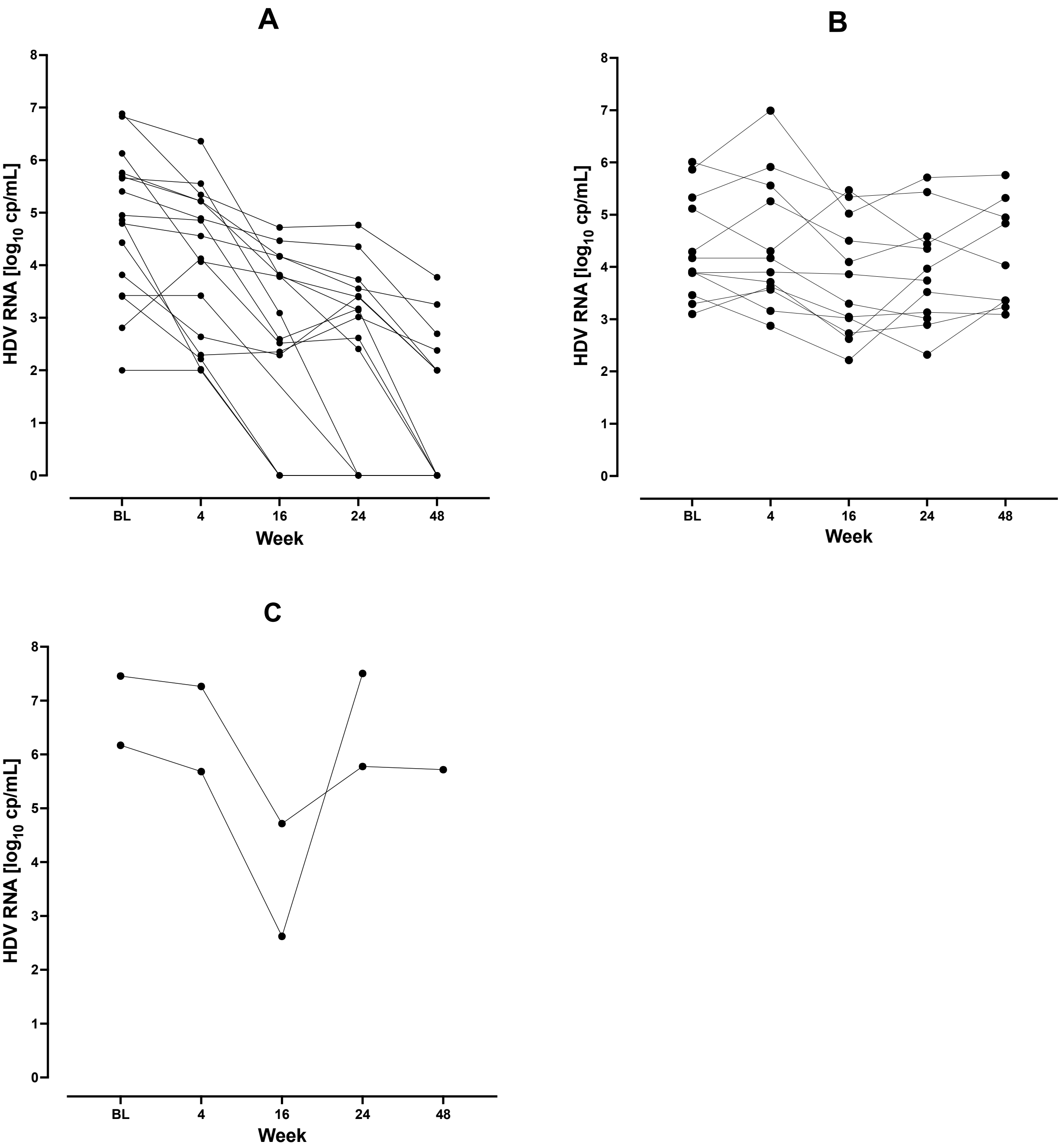

Figure S1: HDV RNA kinetics for (A) 16 virological responders, (B) 12 virological non-responders, and (C) 2 patients with virological breakthrough while receiving bulevirtide treatment at different time points. BL, baseline.

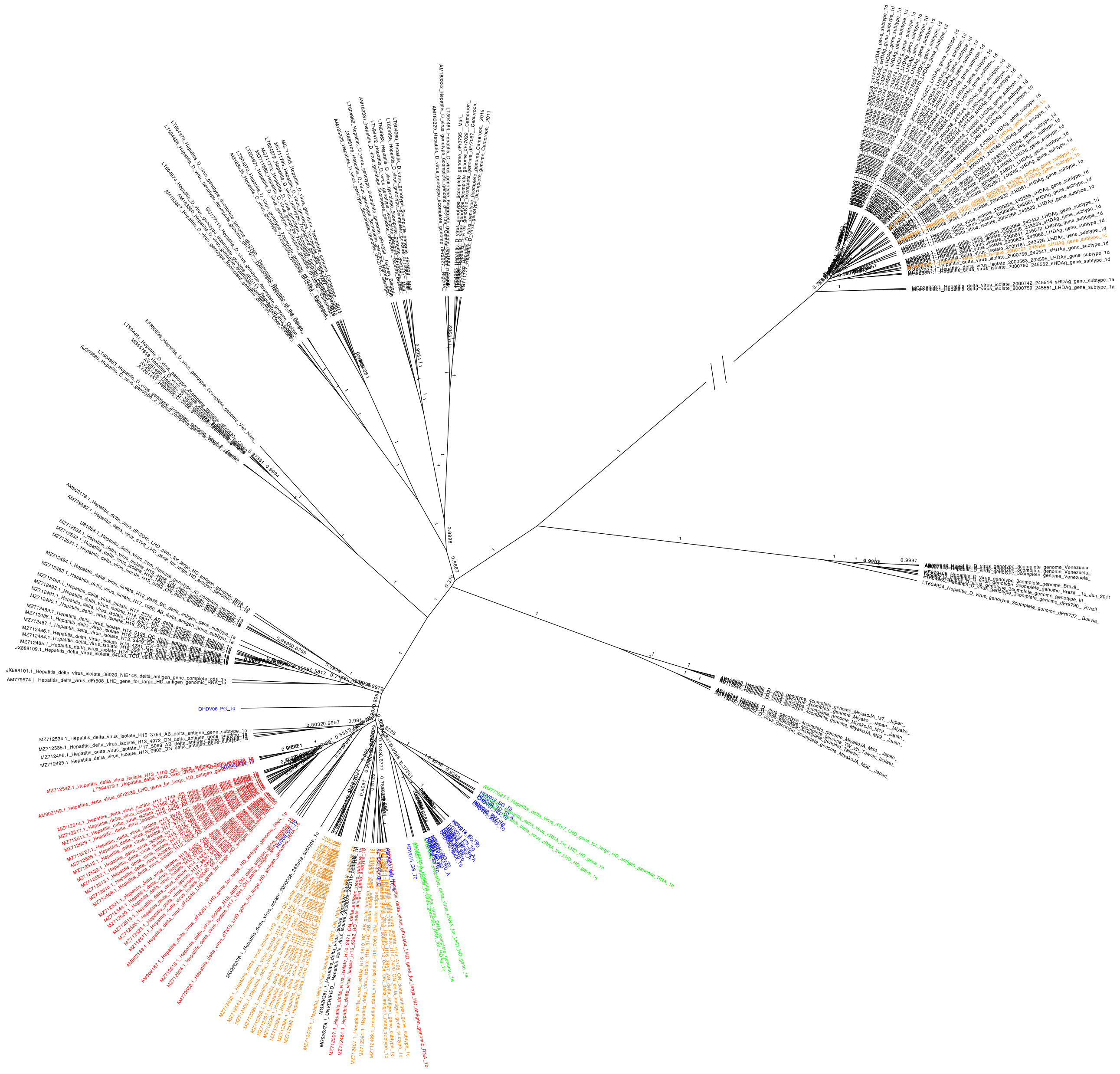

Figure S2: Unrooted phylogenetic tree with non-collapsed lineages. Patient-derived sequences are colored blue, while reference sequences are colored red (subtype 1b), orange (subtype 1c) and green (subtype 1e). The sequences were aligned using MAFFT v7.511. The phylogenetic tree was inferred using BEAST v2.7.3 and Tree Annotator v2.7.3, while tree visualization was implemented with Figtree v1.4.4 and Adobe Illustrator 2025.

Table S1: Characteristics of biochemical and virological variables at baseline and during bulevirtide treatment in virological responder patients.

Parameters	BL	TW4	TW16	TW24	TW48
Age at recruitment, years	55 (49-62)				
Male sex	37.5%				
Body-mass index	24.0 (22.8-27.5)				
Cirrhosis	68.8%				
Previous interferon therapy	68.8%				
Concomitant NUC therapy	100%				
ALT, U/L	82.5 (50.0-103.8)	70.5 (46.0-84.0)	34.5 (25.3-40.5)	25.5 (20.5-32.0)	21.0 (20.0-26.0)
AST, U/L	75.0 (68.5-109.5)	55.0 (47.5-69.8)	37.5 (31.8-51.0)	30.0 (28.0-38.0)	29.0 (27.0-34.0)
Albumin, g/dl	4.2 (4.0-4.4)	4.1 (3.9-4.4)	4.2 (4.0-4.5)	4.3 (4.1-4.4)	4.4 (4.1-4.5)
Bile acids, µmol/L	11.0 (6.6-18.3)	28.5 (15.5-46.2)	36.9 (19.2-45.3)	22.1 (13.9-57.1)	24.9 (17.6-28.6)
Total bilirubin, mg/dL	0.7 (0.7-1.2)	0.8 (0.6-1.1)	0.8 (0.6-1.0)	0.8 (0.7-1.0)	0.8 (0.6-1.2)
Platelet count, x10^3/µL	101.0 (86.3-155.8)	99.5 (86.0-178.8)	98.0 (87.0-184.8)	117.5 (97.8-169.5)	115.0 (93.0-157.0)
HDV RNA, Log cp/mL	5.0 (4.1-5.7)	4.3 (2.6-5.2)	3.1 (2.3-4.0)	3.1 (0.0-3.4)	1.0 (0.0-2.3)
HBV DNA detectable*	43.8%	43.8%	31.3%	25.0%	14.3%
HBsAg, Log IU/mL	3.7 (3.5-4.1)	3.9 (3.6-4.2)	3.8 (3.5-4.2)	3.8 (3.5-4.2)	3.7 (3.3-4.2)
HBcrAg, Log U/mL	3.2 (2.7-4.1)	3.3 (2.5-4.2)	3.2 (2.2-3.9)	3.2 (2.4-3.9)	3.4 (2.6-4.2)
Anti-HBc IgG, COI	39.3 (31.6-47.1)			36.7 (27.6-47.6)	49.9 (39.9-79.9)

* HBV DNA ≥ 10 IU/mL.
Parameter values are either expressed as percentage or median (IQR1 - IQR3).
IQR, interquartile range; NUC, nucleos(t)ide-analogue therapy; ALT, alanine aminotransferase; AST, aspartate aminotransferase; HDV, hepatitis delta virus; HBV, hepatitis B virus; HBsAg, hepatitis B surface antigen; HBcrAg, hepatitis B core-related antigen; anti-HBc IgG, immunoglobulin G antibody to hepatitis B core antigen; COI, Cut-Off-Index.

Table S2: Characteristics of biochemical and virological variables at baseline and during bulevirtide treatment in virological non-responder patients.

Parameters	BL	TW4	TW16	TW24	TW48
Age at recruitment, years	46.5 (40.5-49.5)				
Male sex	57.1%				
Body-mass index	24.0 (22.3-29.3)				
Cirrhosis	85.7%				

Previous interferon therapy	78.6%				
Concomitant NUC therapy	100%				
ALT, U/L	84.0 (55.8-89.8)	62.5 (44.0-84.8)	54.0 (39.3-57.8)	41.5 (33.5-47.8)	58.0 (36.0-64.0)
AST, U/L	68.0 (58.3-83.3)	46.5 (43.3-55.8)	45.0 (33.8-50.8)	38.0 (34.0-46.8)	48.0 (40.0-53.0)
Albumin, g/dl	4.2 (3.9-4.5)	4.3 (3.9-4.5)	4.1 (3.9-4.6)	4.2 (4.1-4.4)	4.3 (4.2-4.5)
Bile acids, µmol/L	7.9 (3.3-13.7)	25.7 (20.7-44.6)	21.7 (16.2-36.8)	35.8 (22.6-52.7)	24.5 (17.8-33.5)
Total bilirubin, mg/dL	1.0 (0.7-1.6)	0.8 (0.7-1.3)	0.9 (0.8-1.4)	0.9 (0.8-1.3)	0.8 (0.6-1.3)
Platelet count, x10^3/µL	124.5 (80.0-183.8)	130.5 (113.5-175.0)	130.5 (94.8-197.0)	129.5 (88.5-187.8)	179.0 (112.0-196.0)
HDV RNA, Log cp/mL	4.3 (3.9-5.9)	4.2 (3.6-5.7)	3.6 (2.8-4.7)	4.2 (3.2-5.2)	4.4 (3.4-5.2)
HBV DNA detectable*	71.4%	71.4%	42.9%	50.0%	70.0%
HBsAg, Log IU/mL	4.1 (3.6-4.2)	4.0 (3.3-4.3)	4.0 (3.5-4.3)	4.0 (3.4-4.3)	4.1 (3.9-4.3)
HBcrAg, Log U/mL	3.5 (2.6-4.0)	3.6 (2.8-4.1)	3.5 (2.9-4.1)	3.6 (2.9-4.2)	3.3 (3.0-4.0)
Anti-HBc IgG, COI	244.7 (127.0-299.4)			222.5 (105.4-300.0)	260.7 (130.0-300.0)

* HBV DNA ≥ 10 IU/mL.

Parameter values are either expressed as percentage or median (IQR1 - IQR3).

IQR, interquartile range; NUC, nucleos(t)ide-analogue therapy; ALT, alanine aminotransferase; AST, aspartate aminotransferase; HDV, hepatitis delta virus; HBV, hepatitis B virus; HBsAg, hepatitis B surface antigen; HBcrAg, hepatitis B core-related antigen; anti-HBc IgG, immunoglobulin G antibody to hepatitis B core antigen; COI, Cut-Off-Index.

Table S3: Summary of baseline polymorphisms detected in the functional domains of delta antigen (HDAg) protein among v-responders and v-non-responders. Corresponding frequencies (number of patients) for each polymorphism and the associated HDV subtype are shown.

Functional demain	v-responders	FREQUENCY (n)	Genotype	Functional demain	v-non-responders	FREQUENCY (n)	Genotype
RBD1	S6T	1	1e	RBD1	S2G	1	1e
	S6K	2	1e		S4A	2	1e
	K7R	1	UND*		**N9S**	1	1e
	**N9S**	4	1b/1e		N9A	1	1e
	N9H	2	1e		**G12S**	1	1e
	N9G	1	UND		V16T	4	1e
	N9V	1	1b		**V16I**	3	1e
	**G12S**	2	1e		**G23A**	4	1e
	G12D	1	1e		**N22S**	1	1e
	E14D	1	1e				
	E15D	1	1b				
	V16L	1	1e				
	**V16I**	3	UND/1e				
	V16F	1	1b				
	Q19K	2	1b				
	**N22S**	5	1b/1e				
	N22V	1	1e				
	N22T	1	UND				
	**G23A**	1	1e				
	G23S	1	1b				
	K25R	1	UND				
	K26E	1	1e				
	K26R	1	1b				
	L27Q	1	UND				
	L27I	1	1b				
	L27A	1	1b				
CCD	D33R	1	1e	CCD	D33E	3	1c/1e
	V37I	1	1e		**V37T**	1	1c/1e
	V37A	1	1e		**E46D**	6	1c/1e
	**V37T**	2	1e		**D47E**	2	1e
	V37E	2	1b				
	K40R	1	1b				
	I41L	1	UND				
	I41V	1	1b				
	K43N	2	1e				
	L44P	1	1b				
	**E46D**	4	1e				
	**D47E**	3	UND/1e				

NLS	**R73K**	2	1e	NLS	**R73K**	1	1e
	T76A	2	1e		**V81I**	1	1e
	**V81I**	2	1e		S83A	1	1e
					R86G	1	1e
					R88K	1	1e
RBD2	**K97Q**	2	1b/1e	RBD2	**K97Q**	1	1e
	K97E	1	1e		**Q100R**	3	1e
	**Q100R**	6	1b/1e		**Q100E**	1	1e
	**Q100E**	1	UND				
RBD3	R139K	1	1e	RBD3	E141K	2	1e
	**V144A**	1	1e		**V144T**	1	1e
VAS	**I198L**	1	1e	VAS	I198K	1	1c
	A202S	2	1b/1e		**I198L**	1	1e
	A202P	1	1b		A202S	2	1c/1e

*UND, GT1 subtype undetermined
Amino acid substitutions that are common between viral responders and viral non-responders are displayed in bold.
RBD, RNA-binding domains; CCD, coiled-coil domain; NLS, nuclear localization sequence; VAS, virus-assembly signal.

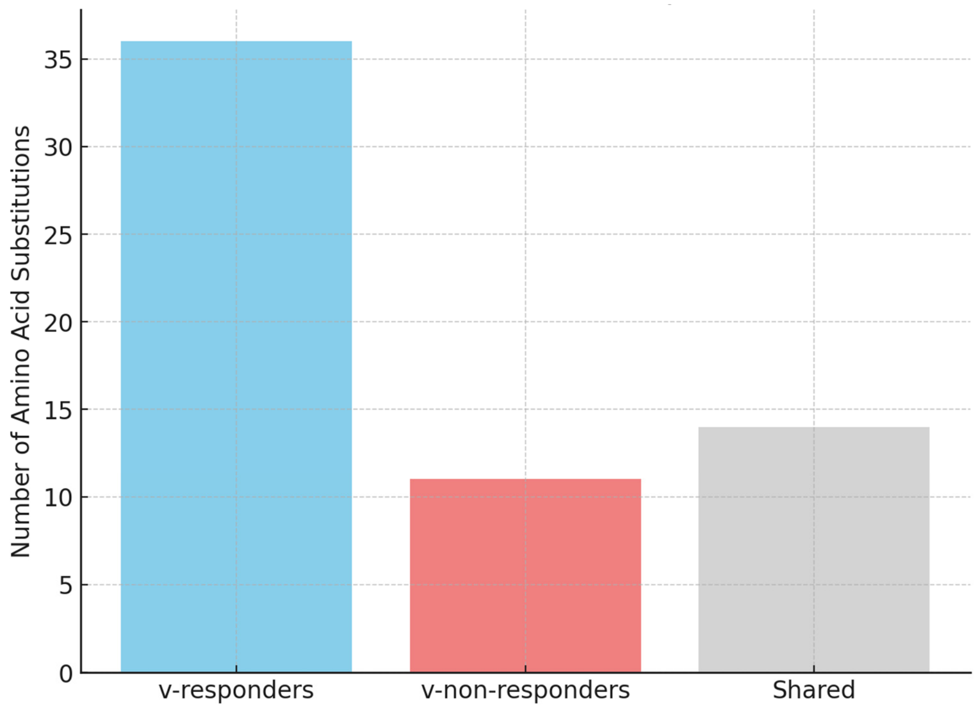

Figure S3: Comparison of amino acid polymorphisms in the functional domains of delta antigen (HDAg) protein among virological responders (v-responders) and virological non-responders (v-non-responders) patients at baseline. The bar graph illustrates the number of amino acid substitutions unique to v-responders (blue), v-non-responders (red), and those shared between the two groups (gray).
